# Ga^3+^ Replacing of Fe^3+^ as a “Trojan Horse” Antimicrobial Strategy: Modeling the Process within Bacterial Catalase–Peroxidase KatG

**DOI:** 10.1021/acs.jcim.6c02504

**Published:** 2026-10-16

**Authors:** Lorenzo Gramolini, Richard López-Corbalán, Jorge García-Millán, Nikoleta Kircheva, Marco Marazzi, Cristina García-Iriepa

**Affiliations:** † 98711 Universidad de Alcalá, Departamento de Química Analítica, Química Física e Ingeniería Química, Functional Molecular Systems (FuMSys) Group, Ctra., Madrid-Barcelona Km. 33,600, E-28805, Alcalá de Henares, Madrid, Spain; ‡ 54525 Institute of Optical Materials and Technologies “Acad. J. Malinowski”, Bulgarian Academy of Sciences, 1113 Sofia, Bulgaria; § University Centre on Tautomeric Research and Education in Science and Technology (ERA Chair UCTREST), University of Plovdiv, 4000 Plovdiv, Bulgaria; ∥ Universidad de Alcalá, Instituto de Investigación Química ″Andrés M. del Río″ (IQAR), Ctra. Madrid-Barcelona Km. 33,600, E-28805, Alcalá de Henares (Madrid), Madrid, Spain

## Abstract

Replacement of Fe^3+^ by Ga^3+^ has emerged as a promising “Trojan horse” antimicrobial strategy in response to the growing threat of antibiotic-resistant bacteria. Because many bacterial proteins hijack host iron to sustain essential redox processes, substitution with redox inactive Ga^3+^, which closely resembles Fe^3+^ in ionic radius but possesses only one oxidation state, can disrupt their activity. Among potential targets, bacterial catalase-peroxidase shows particular sensitivity to Fe^3+^/Ga^3+^ exchange. However, the structural determinants and thermodynamic driving forces governing this substitution remain poorly understood. Here we combine *ad hoc* force field development, molecular dynamics simulations, alchemical free energy calculations, and QM/MM methods to elucidate the molecular basis of Fe^3+^/Ga^3+^ exchange in the bacterial catalase-peroxidase KatG. Our simulations show that the axial coordination environment of the heme B cofactor is largely preserved upon metal substitution, while the distal ligand dynamically alternates between histidine and a coordinated water molecule. Energy decomposition analysis reveals residue specific differences in electrostatic and van der Waals interactions following metal exchange. Free energy calculations indicate that substitution of iron by gallium in the KatG active site is thermodynamically favorable. The presence of a coordinated water molecule emerges as a key structural feature in both metalated states, while distinct binding patterns contribute differently to the overall thermodynamic driving force. These results provide an atomistic description of gallium mediated metal substitution in KatG and offer mechanistic insight into gallium-based antimicrobial strategies, supporting the rational design of new iron-mimetic antibacterial agents.

## Graphical abstract

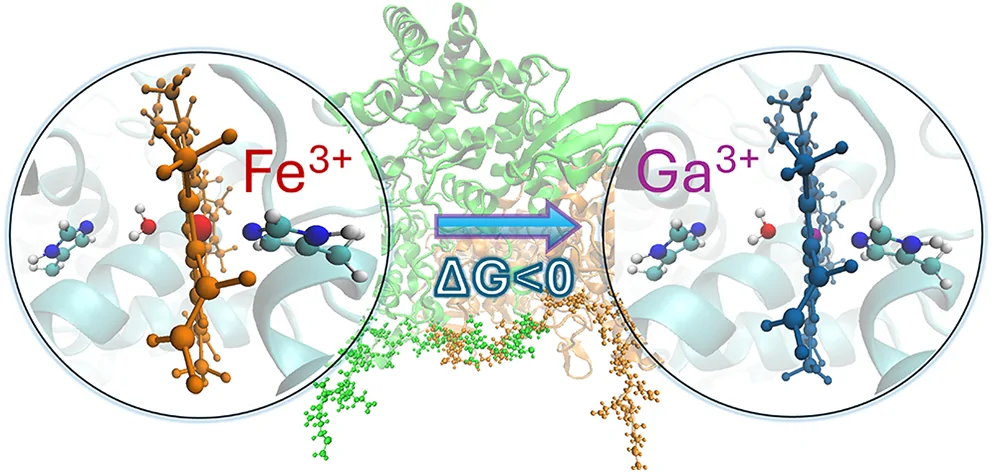

## 1 Introduction

In 1929, Alexander Fleming reported the antibacterial activity of penicillin, inaugurating the antibiotic era and fundamentally transforming modern medicine.^1^ This serendipitous observation not only converted once-lethal infections into treatable conditions but also laid the conceptual and technological foundations for antimicrobial chemotherapy. Nearly a century later, however, the widespread and often indiscriminate use of antibiotics in human health, veterinary practice, and agriculture has accelerated the global emergence of antimicrobial resistance. This evolutionary pressure now threatens to erode the clinical gains initiated by penicillin, undermining routine medical procedures and redefining infectious disease as a major challenge of twenty-first century medicine.^2,3^ In response to this crisis, alternative antimicrobial strategies began to emerge in the late 1990s, including the concept of exploiting bacterial iron acquisition pathways as a “Trojan horse” approach.^4^ In 2007, Kaneko and co-workers demonstrated that gallium disrupts iron-dependent processes in *Pseudomonas aeruginosa*, establishing that Ga^3+^ can mimic Fe^3+^ in biological systems while being unable to undergo redox cycling, thereby impairing essential metabolic functions.^5^ Subsequent studies expanded this framework, positioning gallium as a nonantibiotic siderophore pathway infiltrator and paving the way for the preclinical development of gallium-based therapeutics.^6–8^

Within this context, targeting iron metabolism in *Mycobacterium (M.) tuberculosis* through a Ga^3+^-mediated “Trojan horse” strategy has been shown to affect key iron-dependent enzymes, notably the catalase-peroxidase KatG.^9^ KatG is a homodimeric enzyme in which each ∼80 kDa monomer contains one heme B (Fe^3+^-protoporphyrin IX) cofactor. The dimerization interface contributes to structural stability and optimal positioning of catalytic residues, while each monomer harbors an independent active site (Figure 1).^10^

**Figure 1:**
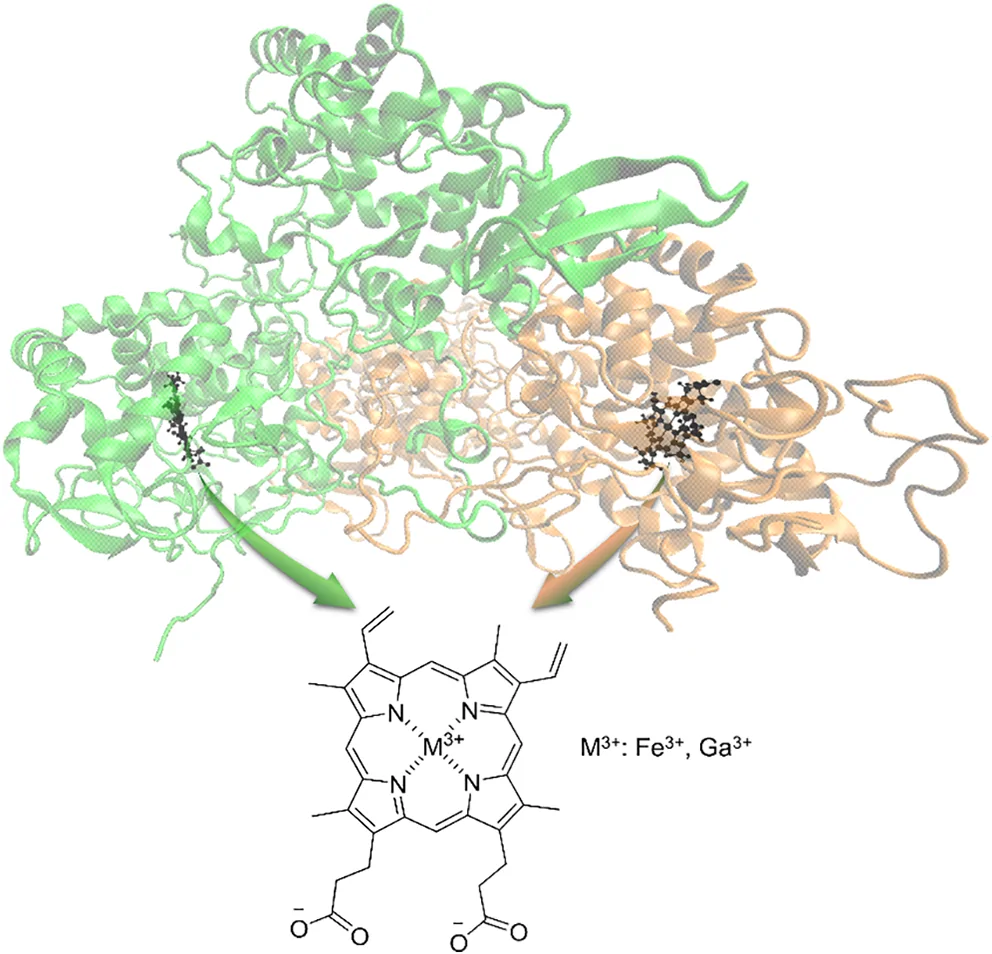
Dimeric bacterial catalase-peroxidase KatG. Structural model of the protein studied (PDB ID: 1SJ2), showing the position of the heme B group in each monomer together with its chemical structure, containing Fe^3+^ (native cofactor) or substituted Ga^3+^ as metal centers.

As its name implies, KatG from *M. tuberculosis* catalyzes two related oxidative reactions that reflect its bifunctional nature: the disproportionation of hydrogen peroxide and the peroxide-dependent oxidation of organic substrates:

$$2H_{2}O_{2}\to 2H_{2}O+O_{2},\left(\mathrm{catalase}\;\mathrm{activity}\right)$$

$$H_{2}O_{2}+{\mathrm{AH}}_{2}\to 2H_{2}O+A,\left(\mathrm{peroxidase}\;\mathrm{activity}\right)$$

where AH_2_ is an electron donor (organic substrate). It should be noted that catalase and peroxidase activities are mechanistically independent and can proceed through parallel pathways, resulting in a bifunctional enzyme, although catalase activity predominates under physiological conditions.^11^ Ga^3+^ scavenging of Fe^3+^ could therefore impair both enzymatic activities, as gallium, unlike iron, cannot support redox cycling owing to its single stable oxidation state, Ga­(III).

Despite a substantial body of biological investigations, comparatively few biochemical studies^12^ and even fewer molecular modeling efforts^13,14^ have addressed the mechanistic basis of the “Trojan horse” strategy and its thermodynamic feasibility. Such information is essential not only to establish the molecular foundations of this antibacterial approach but also to predict which iron dependent enzymes may be more susceptible to Ga^3+^ scavenging, thereby guiding the rational advancement of preclinical development.

From a computational perspective, Kircheva et al. have investigated over this decade the thermodynamics of the Fe^3+^/Ga^3+^ competition in diverse iron-containing active site models^14–17^ including more recently the extracted active site of catalase-peroxidase KatG^18^ to evaluate (i) which Ga^3+^ carrier is most suitable for mediating Fe^3+^ scavenging within the heme B pocket and its protein environment, (ii) the influence of the pH and chemical solvents, and (iii) the thermodynamic driving force of the metal exchange process. Although constituting a relevant step forward, these studies did not account for the electrostatic contribution of the full KatG dimer or the surrounding solvent environment, nor did they consider the dynamic behavior of the system.

Here, we address this gap through an improved multistep computational protocol that extends our previous strategy for ligand-biomolecule interaction targets^19^ enabling the simultaneous characterization of atomistic mechanistic detail and accurate thermodynamic quantities. Specifically, we developed an integrated computational framework combining quantum mechanics (QM), molecular dynamics (MD) simulations, alchemical free energy calculations, and hybrid quantum mechanics/molecular mechanics (QM/MM) approaches to obtain fundamental physicochemical parameters of biological relevance. This strategy reconciles a longstanding methodological challenge in molecular modeling: the integration of high-level quantum mechanical static calculations on chemically representative small models with statistically robust classical dynamical simulations of biologically realistic large systems, while preserving the atomistic resolution required for a chemically meaningful description.

Here we address several key questions: What are the dominant binding modes defining the native KatG active site, and how are they affected by Ga^3+^ replacement of Fe^3+^?^20^ How feasible is Ga^3+^ delivery mediated by protoporphyrin IX? ^21,22^ What thermodynamic driving force governs Ga^3+^ exchange with Fe^3+^-protoporphyrin IX when the latter is tightly bound to KatG, and how do the different binding modes contribute to the overall substitution process?^13^ By combining QM/MM calculations with extensive molecular dynamics simulations, we complement static DFT descriptions with an explicit treatment of protein dynamics. This integrated approach provides mechanistic insight into the potential use of gallium as an abiogenic substitute for iron within the “Trojan horse” antimicrobial strategy.

## 2 Materials and Methods

### 2.1 Quantum Mechanical Description of Heme B with Fe^3+^ and Ga^3+^

The geometries of both Fe^3+^-heme B and Ga^3+^-heme B complexes were optimized by Density Functional Theory (DFT) at the M06–2X^23^/6–31+G­(d,p)^24–27^ level. This approach was previously shown by some of the authors^18^ to reliably reproduce thermodynamic parameters of this class of compounds following validation against B3LYP^28–31^ /6–31+G­(d,p), ωB97X-D^32,33^ /6–31+G­(d,p), and the experiment. The sextet high-spin state was used for Fe^3+^, consistently with electron paramagnetic resonance data^34^ and a recent computational description.^18^ The solvent was treated implicitly by the Solvation Model based on Density (SMD)^35^ designed to accurately describe solvation free energies. For parametrizing the respective force fields, partial atomic charges were calculated by fitting the ElectroStatic Potential (ESP) according to the Merz–Singh–Kollman (MK) scheme.^36,37^ All calculations were performed using the Gaussian16 ^38^ software package.

### 2.2 Molecular Dynamics Simulations

#### 2.2.1 Force Field Parametrization

The force fields of Fe^3+^/Ga^3+^-heme B complexes were parametrized using the MCPB.py code^39^ available in AmberTools22.^40^ This program extracts bond and angle equilibrium values, as well as the corresponding force constants involving the metal center, from the Hessian matrix obtained in the QM frequency calculation of the optimized QM system, according to the Seminario method.^41^ The remaining parameters for the organic ligand were obtained through the Generalized Amber Force Field (GAFF).^42^ The van der Waals parameters for Fe^3+^ and Ga^3+^ were taken from the parametrization of trivalent metal ions by Li et al.^43^ in the Universal Force Field (UFF).^44^ Finally, partial atomic charges were obtained through the REstrained ElectroStatic Potential (RESP)^45^ fitting procedure, employing ESP charges computed at the QM level. The assigned atom types and the force field parameters involving the metal center are shown in Figure S5 and Table S1, respectively. It should be noted that, to avoid biasing the KatG active site toward a particular binding pattern, only nonbonded interactions (i.e., electrostatic and van der Waals contributions) were considered to describe the interactions between the metal and the two residues occupying the axial positions. In contrast, both bonded and nonbonded interactions were included to describe the interaction between the metal and heme B, as the metal is known to be more stable when coordinated within the heme B environment.^46^ Such modeling strategy was already established for several metal ions in different biomolecular environments.^47^ In the specific case of KatG, this means that the generated force fields describe the Fe^3+^/Ga^3+^- heme B encounter complexes, i.e., the preassociation/dissociation intermediates stabilized by noncovalent interactions. This is confirmed by comparison with the available crystallographic data^10,48^ for Fe^3+^/Ga^3+^-heme B and the equilibrium bond lengths and angles involving the metal center obtained from our force fields (Table S1). According to the X-ray data, the Ga^3+^–N distances within heme B are, on average, 0.043 Å shorter than the corresponding Fe^3+^–N distances, which is consistent with the average shortening of 0.053 Å predicted by the equilibrium values of our force fields. N–metal–N and metal–N–N angles do also match the experimental values with an average absolute error of 1.11 ± 0.72 degrees for Fe^3+^ and 0.52 ± 1.02 degrees for Ga^3+^.

#### 2.2.2 Molecular Dynamics Setup and Production Runs

To evaluate the performance of the parametrized force field, four 300 ns trajectories were performed for both Fe^3+^- and Ga^3+^-heme B complexes in explicit water. Moreover, a force field validation using QM optimized geometries as reference is shown in Section S1.2.6. The X-ray crystal structure of *M. tuberculosis* catalase-peroxidase homodimer (PDB ID:1SJ2)^10^ was completed through the AlphaFold^49,50^ software to predict the position of missing residues (see Figure S1 for details). The resulting model was solvated and equilibrated (see Section S1.2.2 of the Supporting Information). Both Fe^3+^ ions of the native dimer were replaced by Ga^3+^ ions to generate a model including two Ga^3+^-heme B complexes. Then, four MD production runs of 300 ns each were performed for both Fe^3+^ and Ga^3+^ models. Furthermore, the equilibrated Fe^3+^ model was used as the starting structure for an additional set of four 300 ns MD simulations of the Ga^3+^ model, and vice versa, resulting in a total simulation time of 2.4 μs per dimer, hence resulting in 4.8 μs sampling for each Fe^3+^/Ga^3+^-heme B active site. Indeed, multiple independent trajectories are recommended to enhance convergence and reproducibility, while initiating simulations from different equilibrated structures helps mitigate potential biases arising from the initial conditions.^51^ It should also be noted that the force fields developed for Fe^3+^/Ga^3+^–heme B allow, in principle, the exploration of any possible binding site for the metal axial ligands. Moreover, to assess protein stability in the absence of cofactors, four 300 ns trajectories were performed for apo-KatG. All the simulations were performed using the GPU version of Amber22.^40^ The calculation of the Root Mean Square Deviations (RMSD) of the active site for each MD trajectory is detailed in Section S4 of the Supporting Information and the corresponding results are shown in Figures S12 and S13. MD based linear interaction energy analyses were performed including a default cutoff of 12 Å to both electrostatic and van der Waals interactions, while periodic boundary conditions and the minimum image convention were enabled.

#### 2.2.3 Alchemical Free Energy Calculations

Taking advantage of the QM-based consistently different force fields of Fe^3+^- and Ga^3+^-heme B complexes, alchemical free energy calculations^52,53^ were carried out to investigate the thermodynamics of substituting Fe^3+^ with Ga^3+^. For each λ window corresponding to an intermediate state, the system was first equilibrated, followed by a 15 ns production run. The production trajectories were then analyzed using the alchemlyb library^54^ to compute the free energy changes (and associated errors) by applying the Thermodynamic Integration (TI) method^55^ and two free energy perturbation more sophisticated variants: Bennett Acceptance Ratio (BAR)^56^ and Multistate Bennett Acceptance Ratio (MBAR).^57^ See Section S1.2.3 of the Supporting Information for details and Section S3 of the Supporting Information, including and Tables S4 and S5, for convergence assessments of the alchemical transformations in the protein (Figures S9) and in explicit water (Figures S10), the neighboring-state overlap (Figure S11), the effective sample size (Table S4), and uncertainty estimates for each individual transformation (Table S5).

### 2.3 Hybrid Quantum Mechanics/Molecular Mechanics Calculations

A representative geometry of each main binding pattern was obtained through clustering analysis (see Section S1.2.4 of the Supporting Information). The Fe^3+^/Ga^3+^-heme B complex and the two axial ligands pointing toward the metal center (either two His side chains or one His side chain and one water molecule) were included in the QM region, while the remaining system was treated at the MM level, interacting electrostatically with the QM region. In the case of the His ligands, the QM/MM boundary was set between

C_α_ (MM) and C_β_ (QM) atoms through the hydrogen link atom scheme, with charge redistribution automatically taken into account as implemented in the Cobramm2.0 software^58^ to avoid overpolarization of the QM region by the neighboring MM charges. The same level of theory used for force field parametrization (M062*X*/6–31+G­(d,p)), resulting from a benchmark performed in a previous study,^18^ was applied to describe the QM region. The force field applied to describe the MM region was the same used for the MD simulations, i.e., ff19SB for the protein region and TIP3P for the water solvent.

The size of the QM region was chosen as to be directly comparable with previous computational studies which neglect the protein explicit environment.^18^ It includes all the residues covalently bound to the metal center and thus was deemed appropriate for the study of the thermodynamics of iron/gallium substitution process. The geometry optimization criteria were the default ones implemented in COBRAMM 2.0 (see Section S1.2.4 in Supporting Information). During geometry optimization of the QM region, the MM region was kept frozen, to facilitate the slow convergence experienced by the QM region within an affordable computational time. The nature of the geometry corresponding to the energy minimum was verified by the absence of imaginary frequencies in a subsequent QM/MM numerical frequency calculation, which also allowed to obtain the QM/MM Gibbs free energy.

We note that using several representative geometries for further QM/MM optimization, or geometries extracted from QM/MM trajectories, could provide a broader exploration of the phase space. However, such an approach would be computationally highly demanding and therefore falls outside the scope of the present work.

The calculations were performed using the Cobramm2.0 software as interface between the QM package (Gaussian16) and the MM package (Amber22).

## 3 Results and Discussion

### 3.1 Structural Effects of Substitution of Fe^3+^ by Ga^3+^ in the Active Site

Extensive MD simulations of the KatG dimer allowed us to define the active site of the iron coordinated heme B (Figure 2). On the distal face of the heme macrocycle, Arg104, Trp107, and His108 (hereafter Hisα) form a tightly packed triad that confirms a motif conserved across catalase-peroxidases and essential for catalytic function.^12,20^ On the proximal face, His270 (hereafter Hisβ) serves as the axial ligand to Fe^3+^, while Trp321 stabilizes the proximal environment through π-stacking interactions with Hisβ.

**Figure 2:**
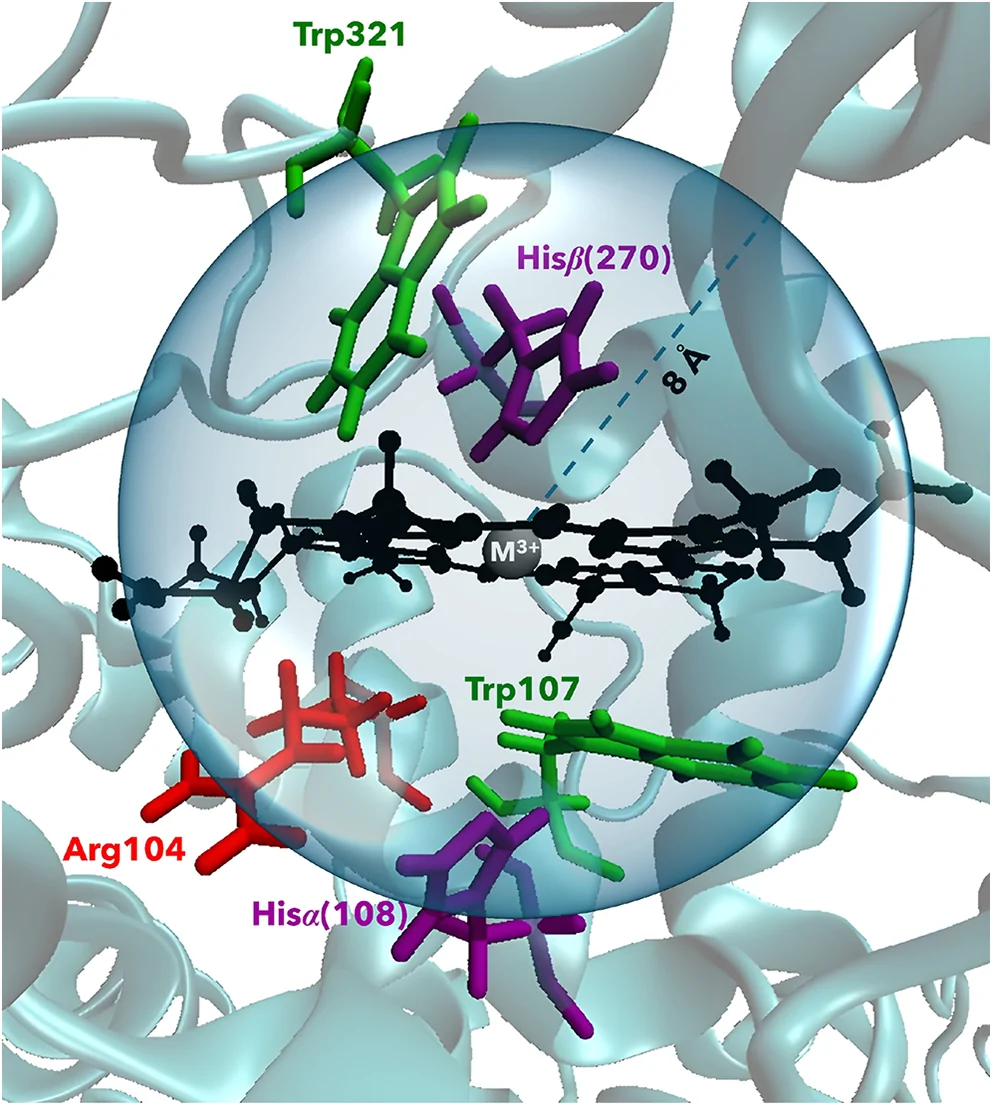
Architecture of the active site. The heme B cofactor is stabilized within the active site by five protein residues located within 8 Å of the metal center (center-of-mass distance). Water molecules are omitted for clarity. Top: proximal side; bottom: distal side.

The distribution of water molecules in the active site reveals the persistence and nature of metal coordination patterns (Figure 3a). In both the native Fe^3+^-heme B and the substituted Ga^3+^-heme B environments, the proximal ligand to the metal center is consistently Hisβ, whereas the distal ligand is predominantly a water molecule (from now on named W–H@Fe/Ga active site), in competition with Hisα (from now on named H–H@Fe/Ga active site), the latter accounting for ca. 26% of the simulated time. As shown in Figure 3b, individual water molecules display an average residence time of approximately 250–300 ns, after which they may be replaced by another water molecule or by Hisα, or occasionally leave the coordination site vacant. This dynamic behavior, typical of water permeable protein pockets^59–61^ is consistent with the catalytic requirements of KatG, as both catalase and peroxidase activities require H_2_O_2_ binding on the distal side of heme B. The distal pocket therefore allows H_2_O_2_ to approach the metal center without displacing a stable ligand, in contrast to the proximal side, where Hisβ remains tightly coordinated. Once bound to heme B, the triad (Arg104, Trp107, Hisα) promotes heterolytic O–O bond cleavage^62^ when Fe^3+^ is present. In particular, Hisα acts as a base that deprotonates the bound H_2_O_2_, thereby facilitating O–O bond cleavage.^63^ When Ga^3+^ replaces Fe^3+^, H_2_O_2_ encounters a very similar binding environment; however, the single stable oxidation state of gallium prevents redox reactivity. Notably, the dominant binding pattern W–H@Fe is consistent with experimental structural data from both X-ray crystallography^12^ and cryo-electron microscopy^20^ which place Hisα approximately 5–6 Å from the heme B iron on the distal side, where it cooperates with Arg104 to stabilize negative charge development during peroxide heterolysis.

**Figure 3:**
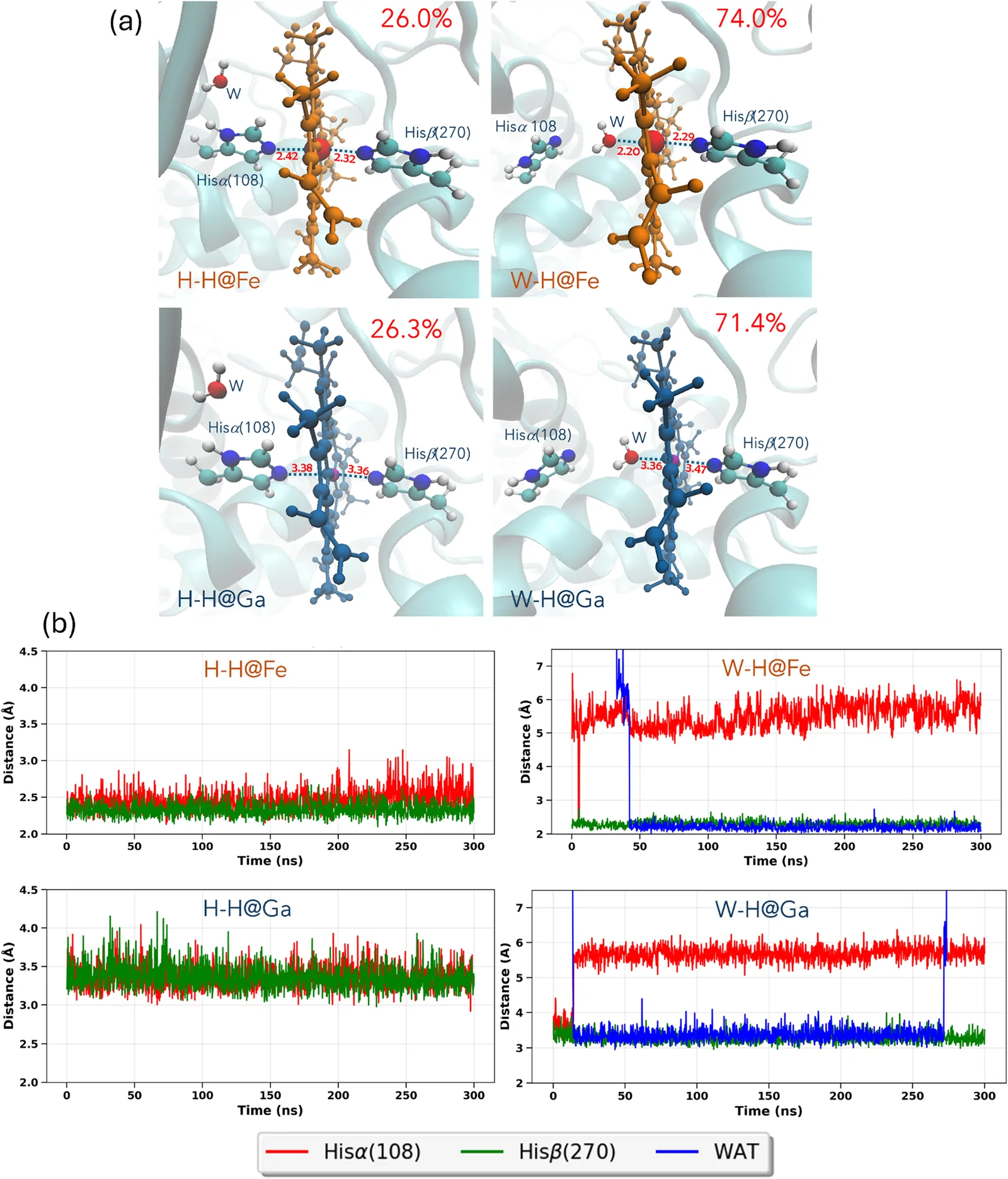
Binding patterns. (a) Representative snapshots showing the main binding modes (percentage relative to total MD simulation time) for Fe^3+^ and Ga^3+^ bound to heme B. The octahedral coordination of both metal centers is completed by axial ligands consisting either of two His side chains (H–H@Fe/Ga) or, more frequently, of one water molecule and one His side chain (W–H@Fe/Ga). Average distances (Å) correspond to the encounter complex preceding chemical coordination. For each binding mode, the proximal side is shown on the right and the distal side on the left. A minor binding pattern is reported in Figure S2. (b) Plot of the corresponding distances.

Only one minor binding pattern (2.3%) was observed in the Ga^3+^-heme B active site (Figure S2). Hence, we can safely state that the KatG active site comprises a heme B cofactor anchored by Hisβ on the proximal side, with the catalytic tetrad positioned on the distal side to facilitate substrate coordination at the metal center. Under physiological conditions, prior to metalated heme B noncovalent substitution and stabilization (i.e., during KatG biosynthesis) Trp321 and Hisβ are expected to largely maintain their structural arrangement, whereas the residues forming the future catalytic tetrad mainly occupy the active site cavity. Upon insertion of the metalated heme B, these residues reorganize to stabilize the cofactor, primarily through hydrophobic interactions (Figure S3).

Because the dominant binding pattern in both iron- and gallium-containing KatG (W–H@Fe/Ga) involves an exchangeable water molecule directly coordinated to the metal center, we analyzed active site hydration through radial distribution functions of the metal–water distances (Figure 4). The results indicate tighter water binding to Fe^3+^ than to Ga^3+^, with the first hydration peak located at 2.60/3.55 Å and a second, exchange-ready water molecule at 4.50/5.75–7.1 Å, suggesting a stronger electrostatic contribution when iron is present. A similar trend, although less pronounced, is observed for the secondary binding pattern H–H@Fe, where a single water molecule within the active site (potentially replacing Hisα) is slightly closer to the metal center and less dispersed in the Fe^3+^ case. The different hydration conditions could also affect the effective availability of H_2_O_2_ as a substrate for initiating catalase or peroxidase activity.^64^ The more prominent presence of an exchangeable water molecule directly coordinated to Fe^3+^, compared with Ga^3+^, should, in principle, decrease the probability that an H_2_O_2_ molecule could coordinate to Ga^3+^ by displacing a water molecule. Indeed, it is known that, in protein environments that are at least partially solvent exposed, water molecules usually form hydrogen bonded networks, with a specific protein bound water molecule coordinated to metal cations having an average residence time in the nanosecond to millisecond time range.^65–68^

**Figure 4:**
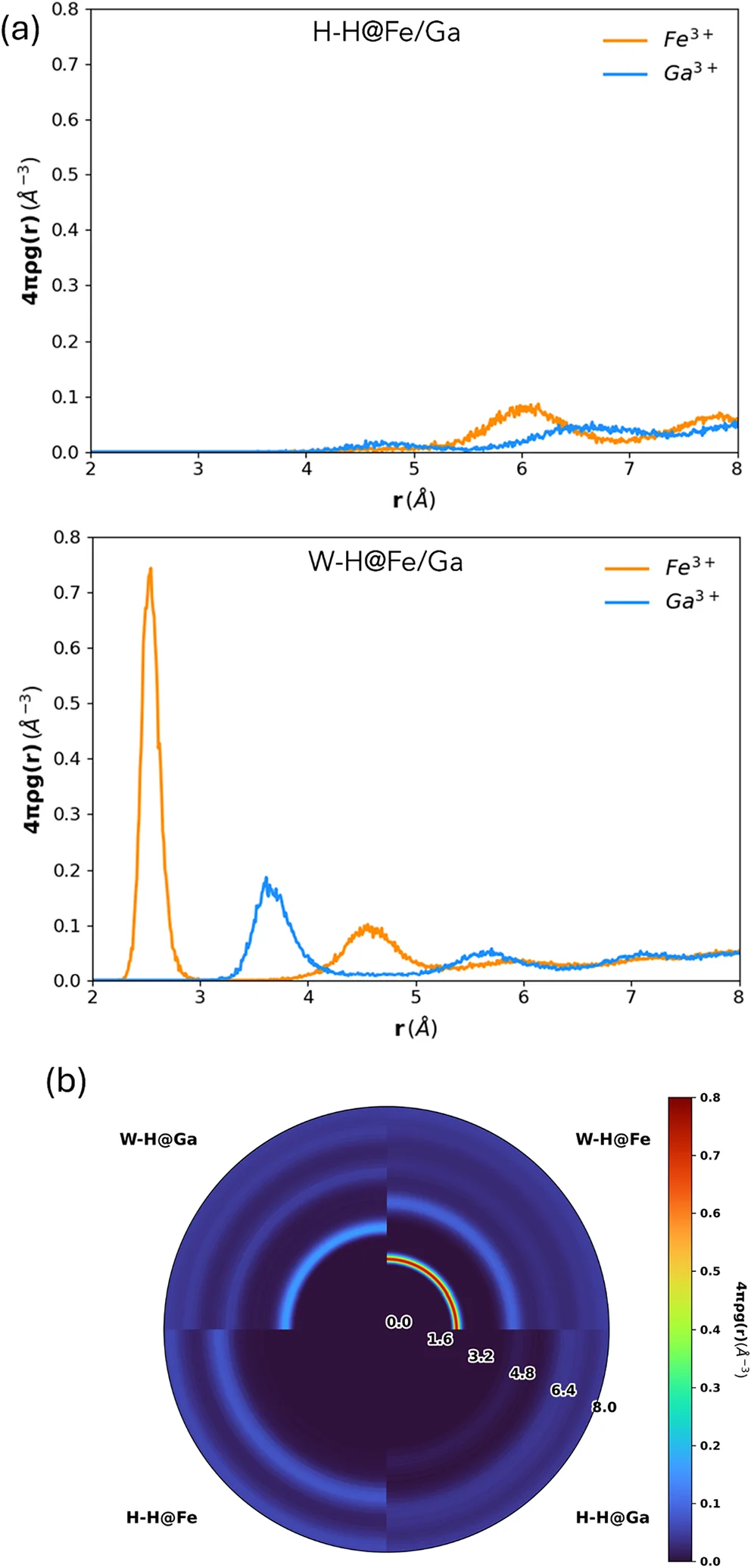
Active site hydration. (a) Radial distribution functions of metal–water distances in the encounter complexes. (b) Top-view heatmap of the same distribution, with each quadrant representing a binding motif. Color intensity delineates coordination-shell boundaries (minima) and highlights the corresponding average metal–water distances (maxima).

Consistent with the water radial distribution functions, linear interaction energy analysis within the active site (Figure 5) shows that electrostatic effects, both stabilizing and destabilizing depending on the specific interaction, are substantially stronger in Fe^3+^-containing KatG than in the Ga^3+^-substituted enzyme. Overall, electrostatic contributions are considerably larger than van der Waals ones. Comparison of the two binding modes (W–H vs H–H) reveals only two quantitative changes on the distal side in terms of electrostatic contributions. The stabilizing interactions Arg104 → heme B and Hisα → Fe^3+^/Ga^3+^ are markedly stronger in the H–H binding mode, supporting the cooperative role of Arg104 and Hisα in stabilizing the negative charge that develops during peroxide heterolysis. Notably, the Arg104 → heme B interaction represents the most stabilizing contribution within the active site. In contrast, the stabilizing interaction Hisβ → Fe^3+^/Ga^3+^ remains essentially unaffected by the binding mode indicating, as previously observed, that the proximal side retains its structural arrangement.

**Figure 5:**
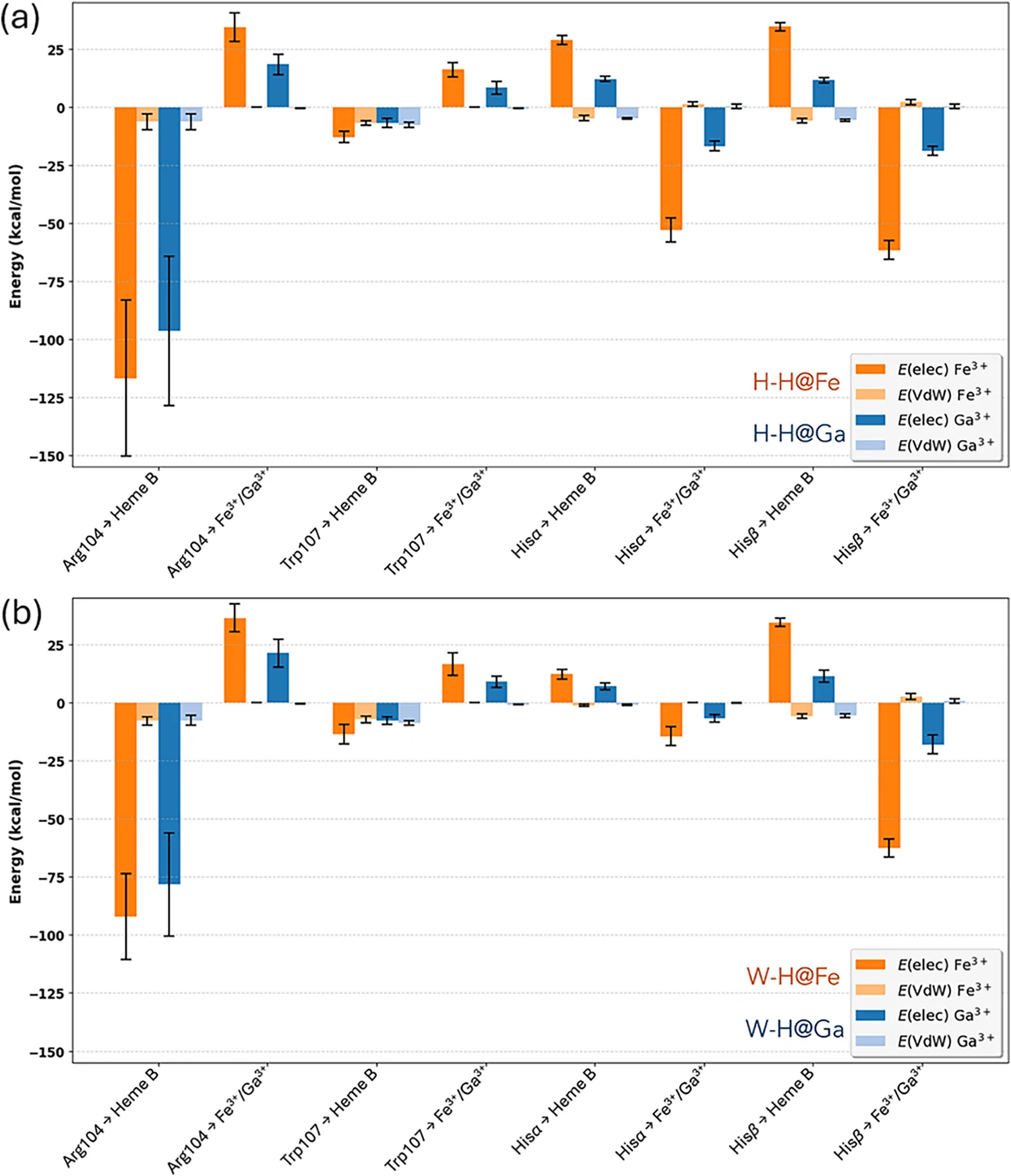
MD based linear interaction energy analysis. Electrostatic and van der Waals contributions are shown for the two binding patterns, H–H@Fe/Ga (a) and W–H@Fe/Ga (b), considering all relevant interactions within the active site. Trp321 is omitted due to negligible contributions. Positive and negative values indicate destabilizing and stabilizing interactions, respectively.

Destabilizing contributions help reveal the overall balance of interactions within the active site. The destabilizing Hisα/Hisβ → heme B interactions indicate that the direct ligands primarily stabilize the metal center, while tending to destabilize the surrounding heme B macrocycle. Conversely, the destabilizing Arg104/Trp107 → Fe^3+^/Ga^3+^ interactions highlight the complementary structural role of residues not directly involved in metal coordination, which instead contribute to stabilizing the heme B framework.

Regarding tryptophan contributions, Trp107 represents the only exception within the active site providing competitive van der Waals interactions that become slightly stronger when gallium replaces iron (Figure 5). This arises from stable π–π stacking with heme B, which is indeed largely driven by van der Waals interactions (Figure S4). This is consistent with experimental observations and with our simulations showing that Trp107 is maintained in a heme B stabilizing position through a unique MYW (Met-Tyr-Trp) adduct in which Tyr229, located slightly farther from the metal center, bridges Met255 and Trp107.^69^ Indeed, although not directly involved in its catalytic activity, the MYW adduct ensures tight retention for H_2_O_2_ disproportionation and peroxidase activity, avoiding heme B release during catalysis.^70,71^

On the proximal side, Trp321 adopts a T shaped orientation relative to heme B (Figure S4) and plays a supportive structural role through π–π stacking with Hisβ, the main proximal ligand to the metal center. Previous studies have shown that Trp321, either alone or in association with Hisβ, contributes to establishing and maintaining the proximal structural arrangement rather than directly interacting with the heme B conjugated system.^13,20,72^

### 3.2 Mechanism and Thermodynamics of the “Trojan Horse” Strategy

Alchemical transformations were employed to determine the energetic feasibility of the “Trojan horse” strategy. As shown in Figure 6, two transformations were modeled: replacement of Fe^3+^ by Ga^3+^ in the solvated KatG dimer (**a**) and the same reaction when both heme B groups are surrounded only by bulk solvent (**b**). This thermodynamic cycle implicitly describes the hypothetical release of metalated heme B from the enzymatic active site into water, either in the Fe-bound form (**c**) or in the Ga-bound form (**d**).

**Figure 6:**
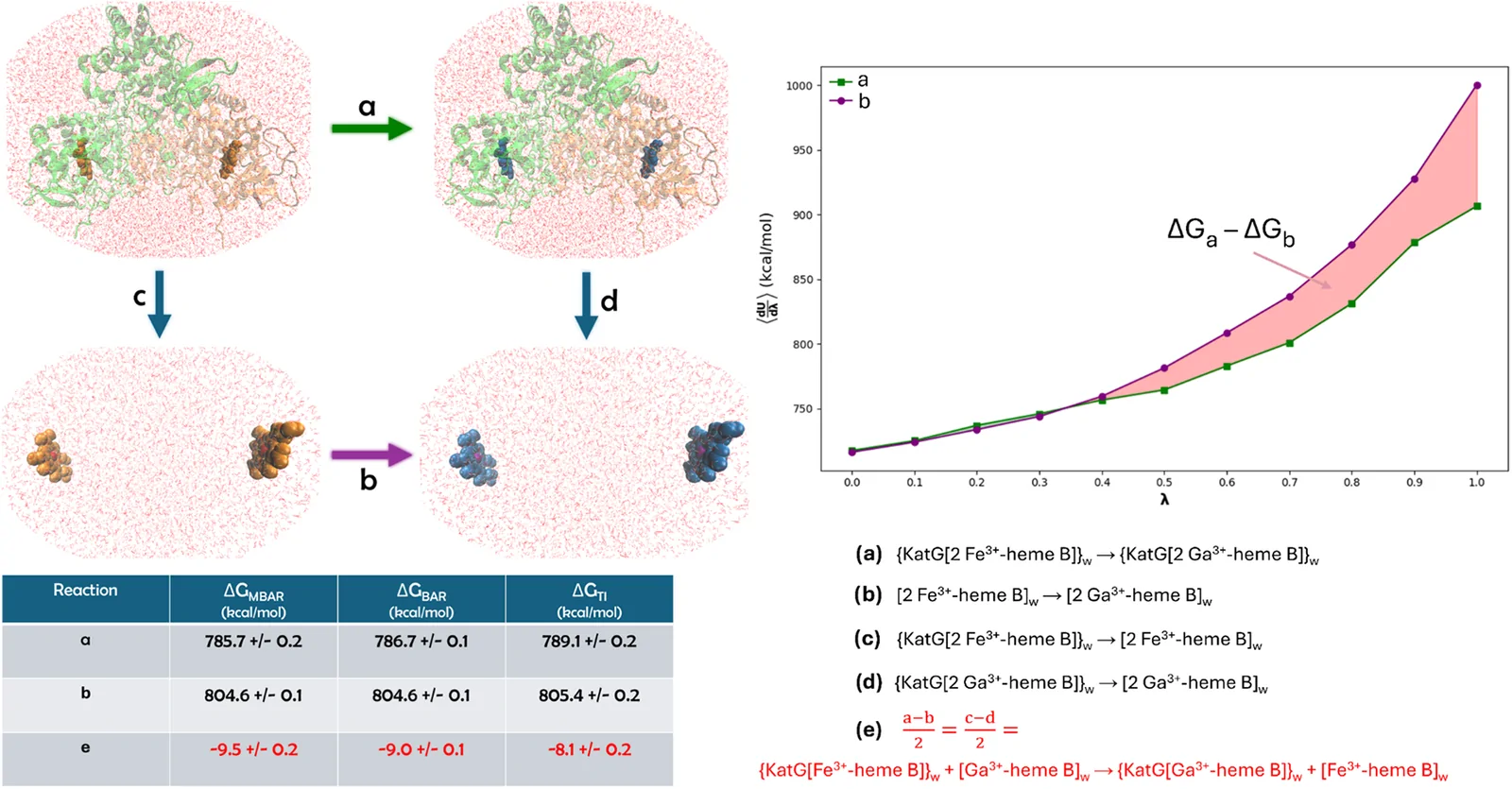
Thermodynamic feasibility of the “Trojan horse” strategy in KatG through MD based alchemical transformation. Reactions **a** (green) and **b** (purple) describe the replacement of Fe^3+^ by Ga^3+^ when heme B is either embedded in the protein or exposed to solvent, respectively. The ΔG difference between these processes is shown as a shaded area, plotted against the coupling parameter λ, which interpolates between initial and final states. Reactions **c** and **d** (blue) correspond to the release of the respective heme B groups with their metal ions. The overall metal replacement in heme B is represented by step **e** (red), obtained as the stoichiometric sum of the preceding reactions. The table summarizes ΔG values for reactions **a**, **b**, and **e** calculated using Thermodynamic Integration (TI) and its variants, Bennett’s Acceptance Ratio (BAR) and Multistate BAR (MBAR).

According to thermodynamic cycle relationships, subtracting reaction **b** from **a** (or equivalently subtracting **d** from **c**) and dividing by two yields the free energy change associated with a single metal substitution. This corresponds to the physically relevant process shown as reaction **e** in Figure 6: Ga^3+^-heme B, one of the forms in which gallium is administered therapeutically^73^ reacts with KatG containing at least one Fe^3+^-heme B cofactor, KatG­[Fe^3+^-heme B], potentially leading to metal exchange. Ga^3+^ is clinically administered as intravenous gallium nitrate, an approved treatment for cancer-related hypercalcemia^74^ however, for catalase-peroxidase enzymes a more effective strategy exploits heme-mimetic gallium porphyrins such as gallium protoporphyrin IX, the metal analogue of heme B.^75^

The calculated free energy change is consistently negative, ranging from −8.1 ± 0.2 to −9.5 ± 0.2 kcal/mol depending on the alchemical transformation approach used (see Materials and Methods Sections 2.2.3 and 1.2.3 of the Supporting Information). The overall Δ*G* can also be visualized as the area between curves depicting **a** and **b** reactions in Figure 6, which represents the variation of the internal energy U along the coupling parameter λ describing the progress of the alchemical transformation (λ = 0 for Fe^3+^ and λ = 1 for Ga^3+^, both in the active site). Because the substitution free energy corresponds to the overall reaction **e** = (**a** **–** **b**)/2, the process is spontaneous (Δ*G* < 0) only if the free energy profile associated with metal substitution in heme B solvated in water (reaction **b**, purple line in the graph of Figure 6) lies above the free energy profile associated with metal substitution within the protein environment (reaction **a**, green line in the graph of Figure 6) along the λ coordinate. Inspection of Figure 6 shows that the two curves nearly overlap up to λ ≈ 0.4, indicating near thermodynamic equilibrium in the early stages of the transformation. Beyond this point, the reaction becomes clearly spontaneous (pink area), reflecting the higher polarity of the aqueous environment relative to the enzymatic active site. The substitution of Fe^3+^ by Ga^3+^ is less destabilized in the protein active site than in water, providing the overall driving force of the process. This behavior can be rationalized by the residual charge on the metal center, which is higher for iron (1.81 *e*) than for gallium (1.04 *e*), leading to stronger electrostatic interactions with the surrounding environment, consistent with the trends observed for the active site binding modes.

Notably, the overall populations of the binding patterns observed during the alchemical transformation within the protein are 77% for W–H and 23% for H–H, in almost quantitative agreement with those obtained from the full MD trajectory (Figure 3a), thereby providing consistency with the MD-derived populations.

Moreover, by accounting for the weighted average of the binding modes identified above (Figures 3a and S2), these calculations can be compared with experimental findings and thus provide indirect support for the physiological relevance of this antimicrobial strategy. Treatment of *M. tuberculosis* with Ga^3+^ tetraphenylporphyrin resulted in a 30% reduction in KatG catalase activity in vitro at 2.5 μg/mL and a 40% reduction at 5 μg/mL, which was directly associated with the accumulation of cytotoxic reactive oxygen species (ROS).^9^ Similar enzymatic inhibition has been experimentally verified in other important pathogens, where treatment with Ga^3+^ compounds reduced catalase activity to ∼40% in *M. abscessus* with 0.16 μg/mL,^76^ and up to 70% in *Pseudomonas aeruginosa*.^77^ Ga^3+^ porphyrins acting as heme B mimetics, including Ga^3+^-protoporphyrin IX and Ga^3+^-mesoporphyrin IX, show also potent growth inhibition against mycobacteria, with Minimum Inhibitory Concentrations (MICs) as low as 0.062 μg/mL for *M. tuberculosis* and 0.5 μg/mL for *M. abscessus* in iron-limited environments.^76^ Hence the efficacy of this “Trojan horse” strategy extends well beyond theoretical models. Experimental studies have proven that Ga^3+^-heme B completely inhibits bacterial growth intracellularly within human macrophage models.^78^ Furthermore, gallium porphyrins exhibit bactericidal activity against multidrug-resistant ESKAPE pathogens in vitro and in murine infection models.^79^

Importantly, the thermodynamic driving force calculated here could help explain the high efficacy of Ga^3+^-heme B observed in experimental competition assays. Indeed, it has been demonstrated in one *Staphylococcus aureus* and in all *Acinetobacter baumannii* that once Ga^3+^-heme B is assimilated by pathogens, even a molar excess of external Fe^3+^-heme B cannot completely abrogate its inhibitory effect.^79^ The favorability of the substitution reaction identified by our calculations may indicate that the metalated cofactor could be retained within the protein environment.

An alternative route to evaluate the thermodynamics of Fe^3+^ replacement by Ga^3+^ is provided by QM/MM free energy calculations. In this framework, the theoretical description is more accurate than in purely MM based alchemical approaches, as it captures not only electrostatic and van der Waals interactions but also covalent contributions. QM/MM free energies are derived from optimized structures and therefore do not include the dynamical information accessible through MD simulations; consequently, the relevant binding configurations identified from the MD trajectories must be examined individually. However, this approach enables direct evaluation of the thermodynamic contribution of each binding mode, thereby providing complementary insight into the “Trojan horse” mechanism.

The thermodynamic cycle shown in Figure 7 describes the equilibrium between binding modes for each metal center (horizontal arrows) and the Fe^3+^/Ga^3+^ substitution within each binding mode (vertical arrows). The replacement of Hisα as metal ligand by a water molecule (H–H → W–H) is strongly favored thermodynamically, particularly for Ga^3+^ (ΔG = −70.0 kcal/mol), in agreement with the MD results (Figure 3). Consistent with the proposed mechanism, Fe^3+^/Ga^3+^ substitution is slightly favorable for the H–H configuration (Δ*G* = −3.5 kcal/mol) and substantially more favorable for the W–H arrangement (Δ*G* = −21.2 kcal/mol). Such a distinction cannot be made when the calculations are performed without explicitly accounting for the environment, considering at the QM level only the metalated heme B and the two ligands coordinated to the metal center (distal and proximal) and treating the surroundings implicitly. In this case, both the H–H@Fe → H–H@Ga and W–H@Fe → W–H@Ga substitution reactions exhibit comparable free energies, ranging from approximately – 15 to – 27 kcal/mol.

**Figure 7:**
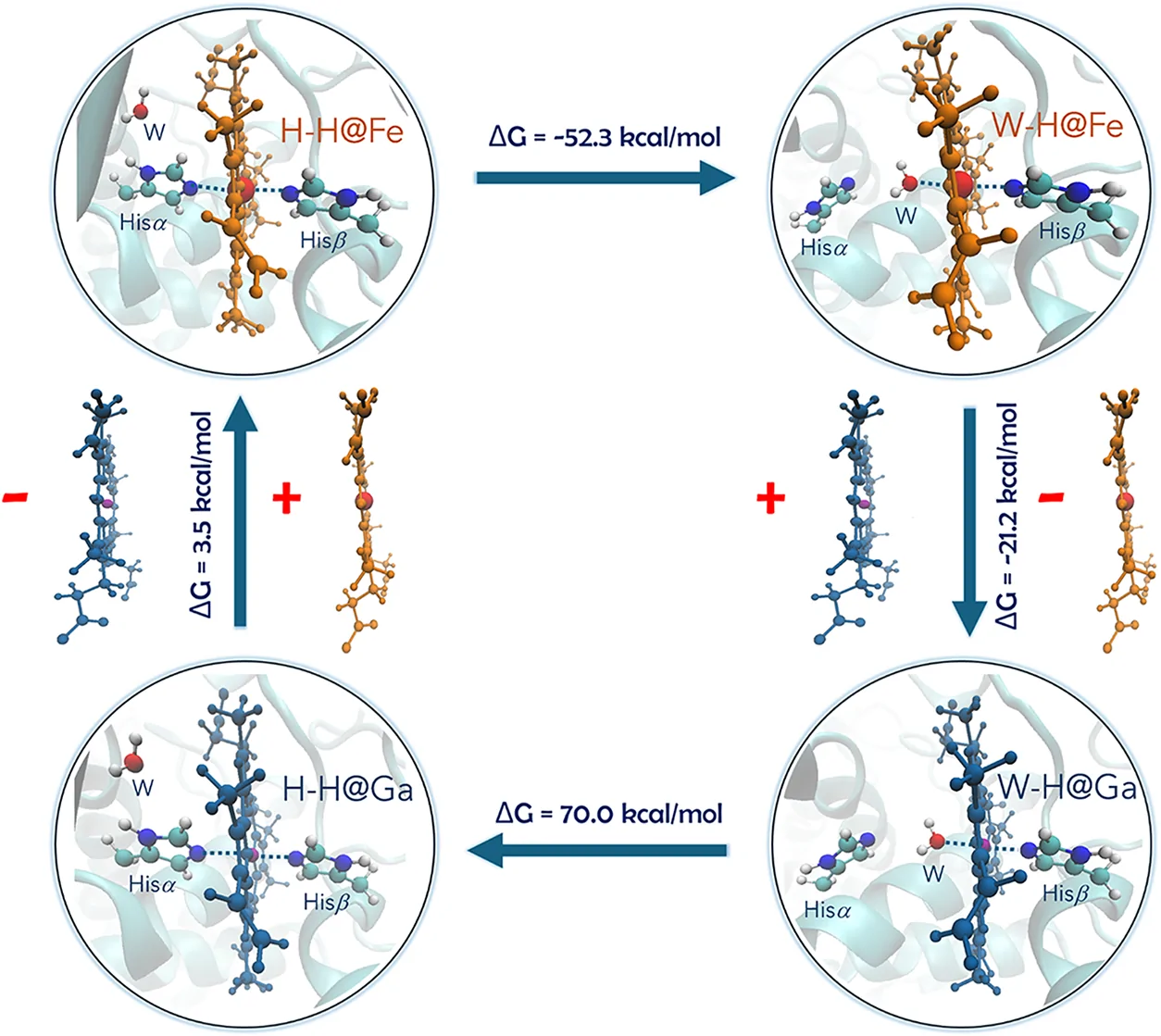
Thermodynamics of the substitution of Fe^3+^ by Ga^3+^ in KatG considering each specific binding mode by QM/MM. Vertical arrows indicate the replacement of the heme B group within the active site by a heme B containing a different metal. Horizontal arrows denote interconversion between binding motifs, involving displacement of a His side chain and the approach of a water molecule, or vice versa. Gibbs free energy changes for each step of the cycle are indicated. The QM region is shown in ball-and-stick representation; MM water molecules are omitted for clarity.

Remarkably, the overall free energy obtained from the alchemical calculations, which accounts for the weighted contribution of all binding modes sampled in MD, lies between these limits (Δ*G* = −8.1 to −9.5 kcal/mol; Figure 6).

For each of the reactions shown in Figure 7, the entropic contribution, −*T*Δ*S*, was calculated (Figure S8). As expected from the greater mobility of the water molecule when it is not coordinated to the metal center, the substitution of water for Hisα as the ligand is associated with a favorable entropic contribution for both metals. However, this contribution to the Gibbs free energy is small (−2.70 and −3.68 kcal/mol for Ga and Fe complexes, respectively), while the largest relative contribution (11%) is observed for the reaction involving the minor binding pattern H–H@Ga → H–H@Fe. For the reaction associated with the most relevant binding pattern, W–H@Fe → W–H@Ga, the entropic contribution is as low as 2.7%. These results indicate that the spontaneity of the thermodynamic process is predominantly enthalpic in nature, with electrostatic interactions between the reactive system and its environment constituting the main driving force, as shown in Figure 5.

## 4 Conclusion

In this work we have investigated the molecular mechanism and thermodynamic feasibility of Ga^3+^ replacing Fe^3+^ in the bacterial catalase-peroxidase KatG, a key component of the gallium based “Trojan horse” antimicrobial strategy. By combining quantum chemical calculations, extensive molecular dynamics simulations, alchemical free energy calculations, and hybrid QM/MM approaches, we provide a consistent atomistic description of the initial and final states determining iron substitution by gallium within the heme B active site and why the process is thermodynamically favored.

Our simulations reveal that the KatG active site exhibits a heterogeneous coordination environment in which the proximal Hisβ residue remains stably coordinated to the metal center, while the distal ligand alternates between Hisα and a coordinated water molecule. The dominant binding motif involves a water molecule on the distal side, highlighting the intrinsic flexibility of the catalytic pocket and its compatibility with substrate access. Importantly, the same structural motifs are maintained upon substitution of Fe^3+^ by Ga^3+^, indicating that gallium can be accommodated within the active site without major structural rearrangements of the protein scaffold.

Energetic decomposition of the interactions within the active site shows that electrostatic contributions dominate the stabilization of the native Fe^3+^ system, whereas their magnitude is significantly reduced when Ga^3+^ replaces iron. This difference reflects the lower effective charge and weaker electrostatic coupling of Ga^3+^ with the surrounding environment, which alters the balance of interactions that stabilize the catalytic metal center. Despite this reduced electrostatic stabilization, the enzyme environment remains compatible with gallium binding due to the cooperative network formed by the distal catalytic residues and the heme macrocycle.

Alchemical free energy calculations demonstrate that the overall Fe^3+^ → Ga^3+^ substitution within the respective encounter complexes in KatG is thermodynamically favorable, with a free energy change of approximately −8 to −9 kcal/mol. The driving force arises from the relative stabilization of Ga^3+^-heme B within the protein environment compared with bulk solvent, supporting the feasibility of the “Trojan horse” mechanism, whereby gallium-containing porphyrins can infiltrate bacterial iron metabolism. Complementary QM/MM calculations further reveal that distinct binding modes contribute differently to this process, with the water-coordinated configuration being strongly predominant over the metal-Hisα configuration, consistently with the structural trends observed in the molecular dynamics’ simulations.

Taken together, these results provide a molecular level description of gallium mediated Fe^3+^ scavenging in KatG, showing how the enzyme can incorporate Ga^3+^ while simultaneously losing catalytic competence due to the redox inactivity of the substituted metal center.

To assess the potential interest of our findings in treating other bacterial pathogens, we identified representative homologues through BLASTp^80,81^ searches against the ClusteredNR database, using the *M. tuberculosis* KatG sequence as a query. Multiple Sequence Alignment (MSA)^82^ analyses (Figures S14–S16) revealed that the five core residues defining the KatG active site architecture are strictly invariant across all analyzed genera, including *Mycobacterium*, *Nocardia*, *Rhodococcus*, *Gordonia*, and *Pseudonocardia*. Notably, His270 and Trp321 exhibited absolute conservation, with no amino acid substitutions detected across the alignment. In any case, although the observed conservation of the KatG active site architecture suggests that the proposed “Trojan horse” strategy may be applicable to a broad range of bacterial pathogens, differences in heme uptake, heme biosynthesis, protein dynamics, cellular iron metabolism, and substrate environment could substantially affect the process and cannot be taken into account by the methodology applied here.

Beyond the present system, the computational pipeline developed here could, in principle, be adapted to the study of metal substitution processes in other metalloproteins, such as Li^+^/Mg^2+^, Ag^+^/Ni^2+^, and Sr^2+^/Ca^2+^.^83^

## Supplementary Material



## Data Availability

Data and Software Availability QM/MM frequency calculations, and MD data of the production runs including initial conditions generated and equilibrated with Fe^3+^, Ga^3+^, Fe^3+^ exchanged with Ga^3+^, and Ga^3+^ exchanged with Fe^3+^, are openly available at 10.5281/zenodo.22850022 The source data for Figure 3a are also openly available at 10.5281/zenodo.19629769

## Notes

Details about the initial structure of the KatG dimer; representative snapshot of the gallium complexed minor binding mode; visualization of the effect of the Fe^3+^-heme B uptake; orientation of tryptophan residues in the active site; details of the quantum mechanical (QM) description of Fe^3+^- and Ga^3+^-heme B complexes; details of the force field parametrization; details of the MD setup and production runs; details of the alchemical free energy calculations; details of the hybrid quantum mechanics/molecular mechanics (QM/MM) calculations; force field validation; comparison between QM and QM/MM Gibbs free energies; entropic contribution; validation of the alchemical transformation; root mean square deviations of the MD production runs; multiple sequence alignment analysis (PDF)

## References

[1] Fleming A. (1929). On the Antibacterial
Action of Cultures of a Penicillium, with Special Reference to Their
Use in the Isolation of B. Influenzae. Br. J.
Exp. Pathol..

[2] Holmes A. H., Moore L. S. P., Sundsfjord A., Steinbakk M., Regmi S., Karkey A., Guerin P. J., Piddock L. J. V. (2016). Understanding
the Mechanisms and Drivers of Antimicrobial Resistance. Lancet.

[3] Murray C. J. L., Ikuta K. S., Sharara F., Swetschinski L., Aguilar G. R., Gray A., Han C., Bisignano C., Rao P., Wool E. (2022). Global
Burden of Bacterial Antimicrobial Resistance
in 2019: A Systematic Analysis. Lancet.

[4] Bernstein L. R. (1998). Mechanisms
of Therapeutic Activity for Gallium. Pharmacol.
Rev..

[5] Kaneko Y., Thoendel M., Olakanmi O., Britigan B. E., Singh P. K. (2007). The Transition
Metal Gallium Disrupts Pseudomonas Aeruginosa Iron Metabolism and
Has Antimicrobial and Antibiofilm Activity. J. Clin. Invest..

[6] Minandri F., Bonchi C., Frangipani E., Imperi F., Visca P. (2014). Promises and
Failures of Gallium as an Antibacterial Agent. Future Microbiol..

[7] Sun W., Qi M., Cheng S., Li C., Dong B., Wang L. (2023). Gallium and
Gallium Compounds: New Insights into the “Trojan Horse”
Strategy in Medical Applications. Mater. Des..

[8] Liu Y., Yang F., Pan Z., Fu Z., Jiang X., Cao Z., Li J., Wang H. (2023). Gallium-Enabled
Bactericidal Medicine. Mater. Today.

[9] Choi S.-r., Kulkarni S., Arnett E., Schlesinger L. S., Britigan B. E., Narayanasamy P. (2024). Efficacy and
Possible Mechanism (s)
of Action of Gallium Tetraphenylporphyrin Nanoparticles against HIV-TB
Coinfection in an in Vitro Granuloma Structure Model. ACS Infect. Dis..

[10] Bertrand T., Eady N. A. J., Jones J. N., Nagy J. M., Jamart-Grégoire B., Raven E. L., Brown K. A. (2004). Crystal Structure of Mycobacterium
Tuberculosis Catalase-Peroxidase. J. Biol. Chem..

[11] Liu A. (2024). Catalase-Peroxidase
(KatG): A Potential Frontier in Tuberculosis Drug Development. Crit. Rev. Biochem. Mol. Biol..

[12] Kapetanaki S. M., Zhao X., Yu S., Magliozzo R. S., Schelvis J. P. M. (2007). Modification of the Active Site of
Mycobacterium Tuberculosis
KatG after Disruption of the Met–Tyr–Trp Cross-Linked
Adduct. J. Inorg. Biochem..

[13] Mo L., Zhang W., Wang J., Weng X. H., Chen S., Shao L. Y., Pang M. Y., Chen Z. W. (2004). Three-Dimensional
Model and Molecular Mechanism of Mycobacterium Tuberculosis Catalase-Peroxidase
(KatG) and Isoniazid-Resistant KatG Mutants. Microb. Drug Resist..

[14] Kircheva N., Dobrev S., Nikolova V., Yocheva L., Angelova S., Dudev T. (2024). Implementation of Three
Gallium-Based Complexes in the “Trojan
Horse” Antibacterial Strategy against A. Baumannii: A DFT Approach. Inorg. Chem..

[15] Kircheva N., Dudev T. (2020). Gallium as an Antibacterial
Agent: A DFT/SMD Study of the Ga3+/Fe3+
Competition for Binding Bacterial Siderophores. Inorg. Chem..

[16] Nikolova V., Angelova S., Markova N., Dudev T. (2016). Gallium as
a Therapeutic
Agent: A Thermodynamic Evaluation of the Competition between Ga3+
and Fe3+ Ions in Metalloproteins. J. Phys. Chem.
B.

[17] Kircheva N., Dobrev S., Petkova V., Yocheva L., Angelova S., Dudev T. (2024). In Silico Analysis of the Ga3+/Fe3+
Competition for Binding the Iron-Scavenging
Siderophores of P. Aeruginosa—Implementation of Three Gallium-Based
Complexes in the “Trojan Horse” Antibacterial Strategy. Biomolecules.

[18] Kircheva N., Angelova S., García-Iriepa C., Marazzi M., Dudev T. (2025). Thermodynamics of the Ga3+/Fe3+ Competition in a Model of the Heme
B-Containing Bacterial Catalase Active Center. Inorg. Chem..

[19] Gramolini L., López-Corbalán R., Marazzi M., García-Iriepa C. (2026). A Fluorescent
Probe under the Microscope Showing Dual Recognition of B-DNA and G-Quadruplex
DNA. Commun. Chem..

[20] Munir A., Wilson M. T., Hardwick S. W., Chirgadze D. Y., Worrall J. A. R., Blundell T. L., Chaplin A. K. (2021). Using Cryo-EM to
Understand Antimycobacterial Resistance in the Catalase-Peroxidase
(KatG) from Mycobacterium Tuberculosis. Structure.

[21] Olakanmi O., Kesavalu B., Pasula R., Abdalla M. Y., Schlesinger L. S., Britigan B. E. (2013). Gallium Nitrate Is Efficacious in Murine Models of
Tuberculosis and Inhibits Key Bacterial Fe-Dependent Enzymes. Antimicrob. Agents Chemother..

[22] Olakanmi O., Britigan B. E., Schlesinger L. S. (2000). Gallium
Disrupts Iron Metabolism
of Mycobacteria Residing within Human Macrophages. Infect. Immun..

[23] Zhao Y., Truhlar D. G. (2008). The M06 Suite of
Density Functionals for Main Group
Thermochemistry, Thermochemical Kinetics, Noncovalent Interactions,
Excited States, and Transition Elements: Two New Functionals and Systematic
Testing of Four M06-Class Functionals and 12 Other Function. Theor. Chem. Acc..

[24] Binkley J. S., Pople J. A., Hehre W. J. (1980). Self-Consistent
Molecular Orbital
Methods. 21. Small Split-Valence Basis Sets for First-Row Elements. J. Am. Chem. Soc..

[25] Gordon M. S., Binkley J. S., Pople J. A., Pietro W. J., Hehre W. J. (1982). Self-Consistent
Molecular-Orbital Methods. 22. Small Split-Valence Basis Sets for
Second-Row Elements. J. Am. Chem. Soc..

[26] Dobbs K. D., Hehre W. J. (1986). Molecular Orbital Theory of the Properties of Inorganic
and Organometallic Compounds 4. Extended Basis Sets for Third-and
Fourth-row, Main-group Elements. J. Comput.
Chem..

[27] Dobbs K. D., Hehre W. J. (1987). Molecular Orbital Theory of the Properties of Inorganic
and Organometallic Compounds 5. Extended Basis Sets for First-row
Transition Metals. J. Comput. Chem..

[28] Becke A. D. (1993). Density-functional
Thermochemistry. III. The Role of Exact Exchange. J. Chem. Phys..

[29] Lee C., Yang W., Parr R. G. (1988). Development
of the Colle-Salvetti
Correlation-Energy Formula into a Functional of the Electron Density. Phys. Rev. B.

[30] Vosko S. H., Wilk L., Nusair M. (1980). Accurate Spin-Dependent Electron
Liquid Correlation Energies for Local Spin Density Calculations: A
Critical Analysis. Can. J. Phys..

[31] Stephens P.
J., Devlin F. J., Chabalowski C. F., Frisch M. J. (1994). Ab Initio Calculation
of Vibrational Absorption and Circular Dichroism Spectra Using Density
Functional Force Fields. J. Phys. Chem. A.

[32] Chai J.-D., Head-Gordon M. (2008). Long-Range
Corrected Hybrid Density Functionals with
Damped Atom–Atom Dispersion Corrections. Phys. Chem. Chem. Phys..

[33] Chai J.-D., Head-Gordon M. (2008). Systematic
Optimization of Long-Range Corrected Hybrid
Density Functionals. J. Chem. Phys..

[34] Torii K., Iizuka T., Ogura Y. (1970). Magnetic Susceptibility
and EPR Measurements
of Catalase and Its Derviatives: A Thermal Equilibrium between the
High-and Low-Spin States in the Catalase-Azide Compound. J. Biochem..

[35] Marenich A. V., Cramer C. J., Truhlar D. G. (2009). Universal
Solvation Model Based on
Solute Electron Density and on a Continuum Model of the Solvent Defined
by the Bulk Dielectric Constant and Atomic Surface Tensions. J. Phys. Chem. B.

[36] Singh U. C., Kollman P. A. (1984). An Approach to Computing
Electrostatic Charges for
Molecules. J. Comput. Chem..

[37] Besler B. H., Merz K. M., Kollman P. A. (1990). Atomic Charges Derived
from Semiempirical Methods. J. Comput. Chem..

[38] Frisch, M. J. ; Trucks, G. W. ; Schlegel, H. B. ; Scuseria, G. E. ; Robb, M. A. ; Cheeseman, J. R. ; Scalmani, G. ; Barone, V. ; Petersson, G. A. ; Nakatsuji, H. ; Li, X. ; Caricato, M. ; Marenich, A. V. ; Bloino, J. ; Janesko, B. G. ; Gomperts, R. ; Mennucci, B. ; Hratchian, H. P. ; Ortiz, J. V. Gaussian 16, Revision C.01., Gaussian, Inc. 2016.

[39] Li P., Merz K. M. (2016). MCPB. Py: A Python Based Metal Center
Parameter Builder. J. Chem. Inf. Model..

[40] Case, D. ; Aktulga, H. ; Belfon, K. ; Ben-Shalom, I. ; Berryman, J. ; Brozell, S. ; Cerutti, D. S. ; Cheatham, I. T. E. ; Cisneros, G. ; Cruzeiro, V. ; Darden, T. ; Duke, R. ; Giambasu, G. ; Gilson, M. ; Gohlke, H. ; Goetz, A. ; Harris, R. ; Izadi, S. ; Izmailov, S. ; Kasavajhala, K. ; Kaymak, M. ; King, E. ; Kovalenko, A. ; Kurtzman, T. ; Lee, T. ; LeGrand, S. ; Li, P. ; Lin, C. ; Liu, J. ; Luchko, T. ; Luo, R. ; Machado, M. ; Man, V. ; Manathunga, M. ; Merz, K. ; Miao, Y. ; Mikhailovskii, O. ; Monard, G. ; Nguyen, H. ; O’Hearn, K. ; Onufriev, A. ; Pan, F. ; Pantano, S. ; Qi, R. ; Rahnamoun, A. ; Roe, D. ; Roitberg, A. ; Sagui, C. ; Schott-Verdugo, S. ; Shajan, A. ; Shen, J. ; Simmerling, C. ; Skrynnikov, N. R. ; Smith, J. ; Swails, J. ; Walker, R. ; Wang, J. ; Wang, J. ; Wei, H. ; Wolf, R. ; Wu, X. ; Xiong, Y. ; Xue, Y. ; York, D. ; Zhao, S. ; Amber, P. K. AMBER 22 Reference Manual; University of California, 2022.

[41] Seminario J. M. (1996). Calculation
of Intramolecular Force Fields from Second-derivative Tensors. Int. J. Quantum Chem..

[42] Wang J., Wolf R. M., Caldwell J. W., Kollman P. A., Case D. A. (2004). Development
and Testing of a General Amber Force Field. J. Comput. Chem..

[43] Li Z., Song L. F., Li P., Merz K. M. (2021). Parametrization of Trivalent and
Tetravalent Metal Ions for the OPC3,
OPC, TIP3P-FB, and TIP4P-FB Water Models. J.
Chem. Theory Comput..

[44] Rappé A. K., Casewit C. J., Colwell K. S., Goddard W. A., Skiff W. M. (1992). UFF, a Full Periodic
Table Force Field for Molecular
Mechanics and Molecular Dynamics Simulations. J. Am. Chem. Soc..

[45] Bayly C. I., Cieplak P., Cornell W., Kollman P. A. (1993). A Well-Behaved Electrostatic
Potential Based Method Using Charge Restraints for Deriving Atomic
Charges: The RESP Model. J. Phys. Chem. A.

[46] Oda A., Yamaotsu N., Hirono S. (2005). New AMBER Force Field Parameters
of Heme Iron for Cytochrome P450s Determined by Quantum Chemical Calculations
of Simplified Models. J. Comput. Chem..

[47] Li P., Merz K. M. (2017). Metal Ion Modeling Using Classical
Mechanics. Chem. Rev..

[48] Dodd E. L., Bohle D. S. (2014). Orienting the Heterocyclic
Periphery: A Structural
Model for Chloroquine’s Antimalarial Activity. Chem. Commun..

[49] Jumper J., Evans R., Pritzel A., Green T., Figurnov M., Ronneberger O., Tunyasuvunakool K., Bates R., Žídek A., Potapenko A. (2021). Highly Accurate Protein Structure Prediction
with AlphaFold. Nature.

[50] Fleming J., Magana P., Nair S., Tsenkov M., Bertoni D., Pidruchna I., Lima Afonso M. Q., Midlik A., Paramval U., Žídek A., Laydon A., Kovalevskiy O., Pan J., Cheng J., Avsec Ž., Bycroft C., Wong L. H., Last M., Mirdita M., Steinegger M., Kohli P., Váradi M., Velankar S. (2025). AlphaFold Protein Structure
Database and 3D-Beacons: New Data and Capabilities. J. Mol. Biol..

[51] Ormeño F., General I. J. (2024). Convergence and
Equilibrium in Molecular Dynamics Simulations. Commun. Chem..

[52] York D. M. (2023). Modern
Alchemical Free Energy Methods for Drug Discovery Explained. ACS Phys. Chem. Au.

[53] Gumbart J. C., Roux B., Chipot C. (2013). Standard Binding
Free Energies from
Computer Simulations: What Is the Best Strategy?. J. Chem. Theory Comput..

[54] Wu Z., Dotson D. L., Alibay I., Allen B. K., Barhaghi M. S., Hénin J., Joseph T. T., Kenney I. M., Lee H., Li H. (2024). Alchemlyb: The Simple Alchemistry Library. J. Open Source Software.

[55] Kollman P. (1993). Free Energy
Calculations: Applications to Chemical and Biochemical Phenomena. Chem. Rev..

[56] Bennett C. H. (1976). Efficient
Estimation of Free Energy Differences from Monte Carlo Data. J. Comput. Phys..

[57] Shirts M. R., Chodera J. D. (2008). Statistically Optimal
Analysis of Samples from Multiple
Equilibrium States. J. Chem. Phys..

[58] Weingart O., Nenov A., Altoè P., Rivalta I., Segarra-Martí J., Dokukina I., Garavelli M. (2018). COBRAMM 2.0—A Software Interface
for Tailoring Molecular Electronic Structure Calculations and Running
Nanoscale (QM/MM) Simulations. J. Mol. Model..

[59] Guo Y., Beyle F. E., Bold B. M., Watanabe H. C., Koslowski A., Thiel W., Hegemann P., Marazzi M., Elstner M. (2016). Active Site
Structure and Absorption Spectrum of Channelrhodopsin-2 Wild-Type
and C128T Mutant. Chem. Sci..

[60] Schiebel J., Gaspari R., Wulsdorf T., Ngo K., Sohn C., Schrader T. E., Cavalli A., Ostermann A., Heine A., Klebe G. (2018). Intriguing Role of Water in Protein-Ligand
Binding Studied by Neutron Crystallography on Trypsin Complexes. Nat. Commun..

[61] Pantsar T., Kaiser P. D., Kudolo M., Forster M., Rothbauer U., Laufer S. A. (2022). Decisive Role of Water and Protein
Dynamics in Residence
Time of P38α MAP Kinase Inhibitors. Nat.
Commun..

[62] Zhao X., Yu H., Yu S., Wang F., Sacchettini J. C., Magliozzo R. S. (2006). Hydrogen Peroxide-Mediated Isoniazid
Activation Catalyzed
by Mycobacterium Tuberculosis Catalase–Peroxidase (KatG) and
Its S315T Mutant. Biochemistry.

[63] Heering H.
A., Indiani C., Regelsberger G., Jakopitsch C., Obinger C., Smulevich G. (2002). New Insights
into the Heme Cavity
Structure of Catalase-Peroxidase: A Spectroscopic Approach to the
Recombinant Synechocystis Enzyme and Selected Distal Cavity Mutants. Biochemistry.

[64] Carpena X., Loprasert S., Mongkolsuk S., Switala J., Loewen P. C., Fita I. (2003). Catalase-Peroxidase
KatG of Burkholderia Pseudomallei at 1.7Å
Resolution. J. Mol. Biol..

[65] Stachura M., Chakraborty S., Gottberg A., Ruckthong L., Pecoraro V. L., Hemmingsen L. (2017). Direct Observation
of Nanosecond
Water Exchange Dynamics at a Protein Metal Site. J. Am. Chem. Soc..

[66] Rocchi C., Rita Bizzarri A., Cannistraro S. (1997). Water Residence Times around Copper
Plastocyanin: A Molecular Dynamics Simulation Approach. Chem. Phys..

[67] Kaieda S., Halle B. (2013). Internal Water and Microsecond Dynamics
in Myoglobin. J. Phys. Chem. B.

[68] Bertini I., Luchinat C., Nerinovski K., Parigi G., Cross M., Xiao Z., Wedd A. G. (2003). Application
of NMRD to Hydration
of Rubredoxin and a Variant Containing a (Cys-S)­3FeIII­(OH) Site. Biophys. J..

[69] Singh R., Switala J., Loewen P. C., Ivancich A. (2007). Two [Fe­(IV)­O
Trp•]
Intermediates in M. Tuberculosis Catalase-Peroxidase Discriminated
by Multifrequency (9–285 GHz) EPR Spectroscopy: Reactivity
toward Isoniazid. J. Am. Chem. Soc..

[70] Zhao X., Yu S., Ranguelova K., Suarez J., Metlitsky L., Schelvis J. P. M., Magliozzo R. S. (2009). Role of
the Oxyferrous Heme Intermediate
and Distal Side Adduct Radical in the Catalase Activity of Mycobacterium
Tuberculosis KatG Revealed by the W107F Mutant *. J. Biol. Chem..

[71] Zhao X., Suarez J., Khajo A., Yu S., Metlitsky L., Magliozzo R. S. (2010). A Radical on the Met-Tyr-Trp Modification Required
for Catalase Activity in Catalase-Peroxidase Is Established by Isotopic
Labeling and Site-Directed Mutagenesis. J. Am.
Chem. Soc..

[72] Reyes Y. I. A., Franco F. C. (2019). DFT Study on the Effect of Proximal Residues on the
Mycobacterium Tuberculosis Catalase-Peroxidase (KatG) Heme Compound
I Intermediate and Its Bonding Interaction with Isoniazid. Phys. Chem. Chem. Phys..

[73] Kurtuldu F., Mutlu N., Boccaccini A. R., Galusek D. (2022). Gallium Containing
Bioactive Materials: A Review of Anticancer, Antibacterial, and Osteogenic
Properties. Bioact. Mater..

[74] Warrell R. P., Israel R., Frisone M., Snyder T., Gaynor J. J., Bockman R. S. (1988). Gallium Nitrate
for Acute Treatment of Cancer-Related
Hypercalcemia. Ann. Int. Med..

[75] Hijazi S., Visca P., Frangipani E. (2017). Gallium-Protoporphyrin IX Inhibits
Pseudomonas Aeruginosa Growth by Targeting Cytochromes. Front. Cell. Infect. Microbiol..

[76] Choi S.-r., Switzer B., Britigan B. E., Narayanasamy P. (2020). Gallium Porphyrin
and Gallium Nitrate Synergistically Inhibit Mycobacterial Species
by Targeting Different Aspects of Iron/Heme Metabolism. ACS Infect. Dis..

[77] Scott Z. W., Choi S., Britigan B. E., Narayanasamy P. (2023). Development
of Gallium­(III) as an Antimicrobial Drug Targeting Pathophysiologic
Iron Metabolism of Human Pathogens. ACS Infect.
Dis..

[78] Abdalla M. Y., Switzer B. L., Goss C. H., Aitken M. L., Singh P. K., Britigan B. E. (2015). Gallium Compounds Exhibit Potential
as New Therapeutic
Agents against Mycobacterium Abscessus. Antimicrob.
Agents Chemother..

[79] Hijazi S., Visaggio D., Pirolo M., Frangipani E., Bernstein L., Visca P. (2018). Antimicrobial Activity of Gallium
Compounds on ESKAPE Pathogens. Front. Cell.
Infect. Microbiol..

[80] Altschul S. F., Madden T. L., Schäffer A. A., Zhang J., Zhang Z., Miller W., Lipman D. J. (1997). Gapped
BLAST and PSI-BLAST: A New
Generation of Protein Database Search Programs. Nucleic Acids Res..

[81] Altschul S. F., Gish W., Miller W., Myers E. W., Lipman D. J. (1990). Basic Local
Alignment Search Tool. J. Mol. Biol..

[82] Agarwala R., Barrett T., Beck J. (2018). Database Resources of
the National Center for Biotechnology Information. Nucleic Acids Res..

[83] Smethurst D. G. J., Shcherbik N. (2021). Interchangeable
Utilization of Metals: New Perspectives
on the Impacts of Metal Ions Employed in Ancient and Extant Biomolecules. J. Biol. Chem..

