# Supplementary material for: Ga3+ Replacing of Fe3+ as a “Trojan Horse” Antimicrobial Strategy: Modeling the Process within Bacterial Catalase–Peroxidase KatG
Source: J Chem Inf Model. 2026 Oct 16;66(21):14102–14. doi: 10.1021/acs.jcim.6c02504 (PMC13753714; doi:10.1021/acs.jcim.6c02504)
Supplement: Supplementary file 1 [file ci6c02504_si_001.pdf]

Supporting Information

Ga^{3+} Replacing of Fe^{3+} as a 'Trojan Horse' Antimicrobial Strategy: Modeling the Process within Bacterial Catalase–Peroxidase KatG

Lorenzo Gramolini,[1] Richard López-Corbalán,[1] Jorge García-Millán,[1] Nikoleta Kircheva,[2,3] Marco Marazzi,*[1,4] Cristina García-Iriepa*[1,4]

[1] Universidad de Alcalá, Departamento de Química Analítica, Química Física e Ingeniería Química, Functional Molecular Systems (FuMSys) group, Ctra. Madrid-Barcelona Km. 33,600, E-28805, Alcalá de Henares (Madrid), Spain

[2] Institute of Optical Materials and Technologies "Acad. J. Malinowski", Bulgarian Academy of Sciences, 1113 Sofia, Bulgaria

[3] University Centre on Tautomeric Research and Education in Science and Technology (ERA Chair UCTREST), University of Plovdiv, Plovdiv 4000, Bulgaria

[4] Universidad de Alcalá, Instituto de Investigación Química "Andrés M. del Río" (IQAR), Ctra. Madrid-Barcelona Km. 33,600, E-28805, Alcalá de Henares (Madrid), Spain

* Corresponding author email: marco.marazzi@uah.es; cristina.garciai@uah.es

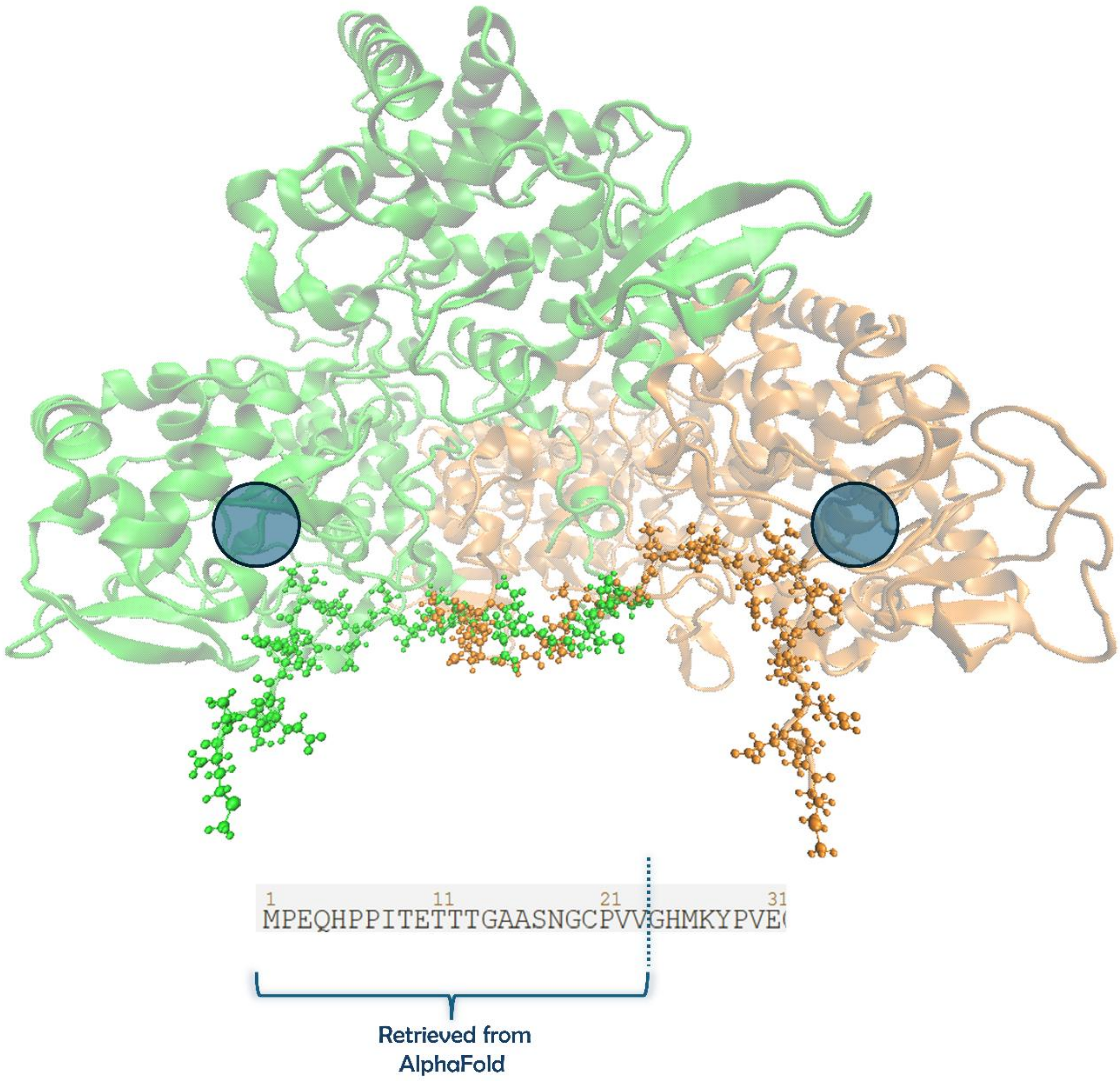

Figure S1. Initial structure of the KatG dimer. Heavy-atom coordinates were obtained from the crystal structure (PDB ID: 1SJ2; X-ray diffraction, 2.41 Å resolution). Missing residues (residues 1 to 23 in each monomer from the N-terminus; ball-and-stick representation) in both homodimers were completed by superposition with AlphaFold's prediction AF-P9WIE5-F1[1] and correspond to flexible unstructured regions characterized by pLDDT scores < 50. Since such regions extend outward from the monomer-monomer interaction interface directly into the solvent bulk, they do not affect the active sites (highlighted in blue).

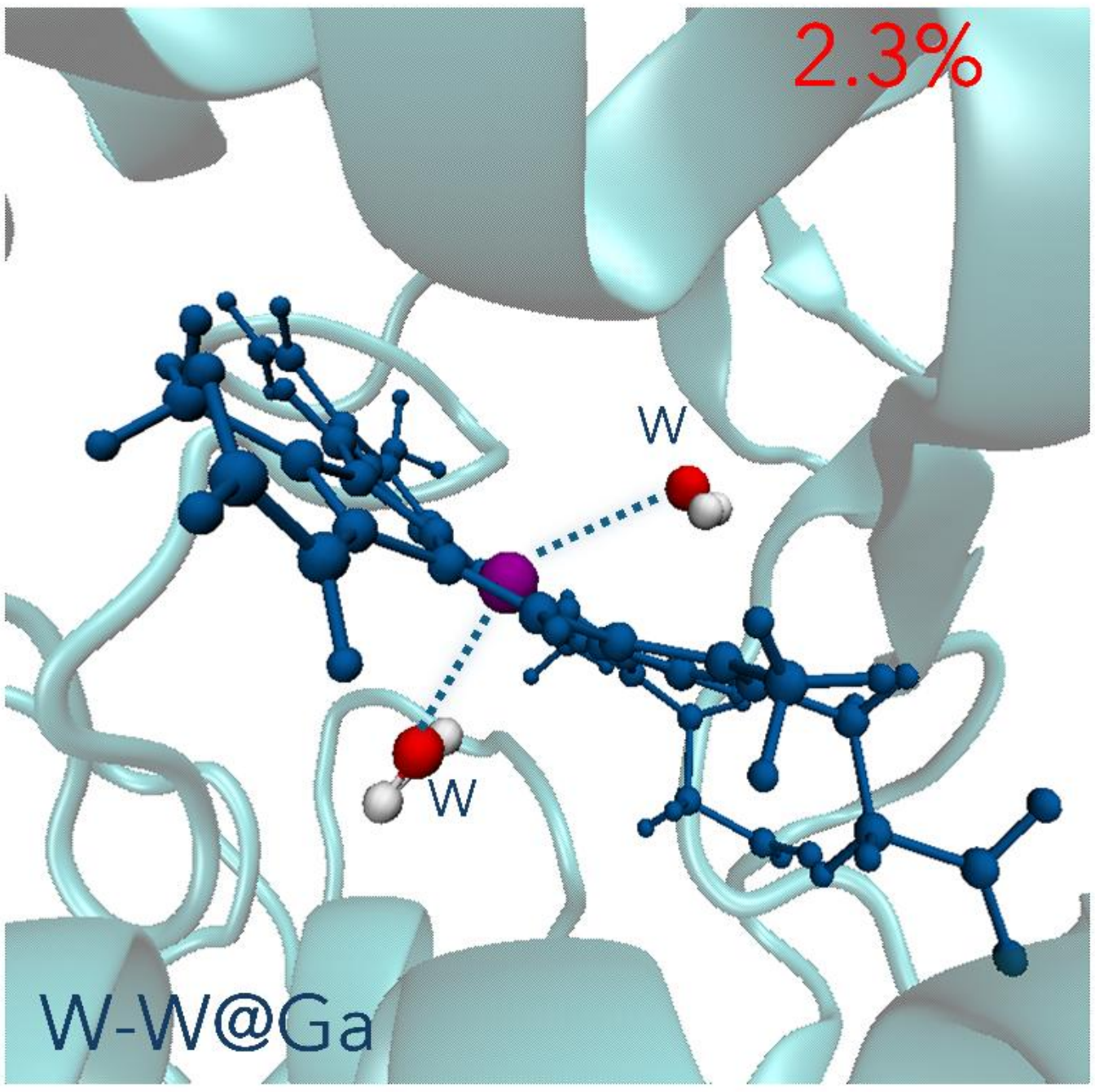

Figure S2. Minor W-W@Ga binding pattern. Representative snapshot of the minor binding mode (percentage relative to the total MD simulation time). Upon replacement of Fe^{3+} by Ga^{3+} in heme B, the proximal Hisβ can be substituted by a water molecule, while a second water molecule coordinates the metal on the distal side, resulting in two water molecules bound to Ga^{3+}.

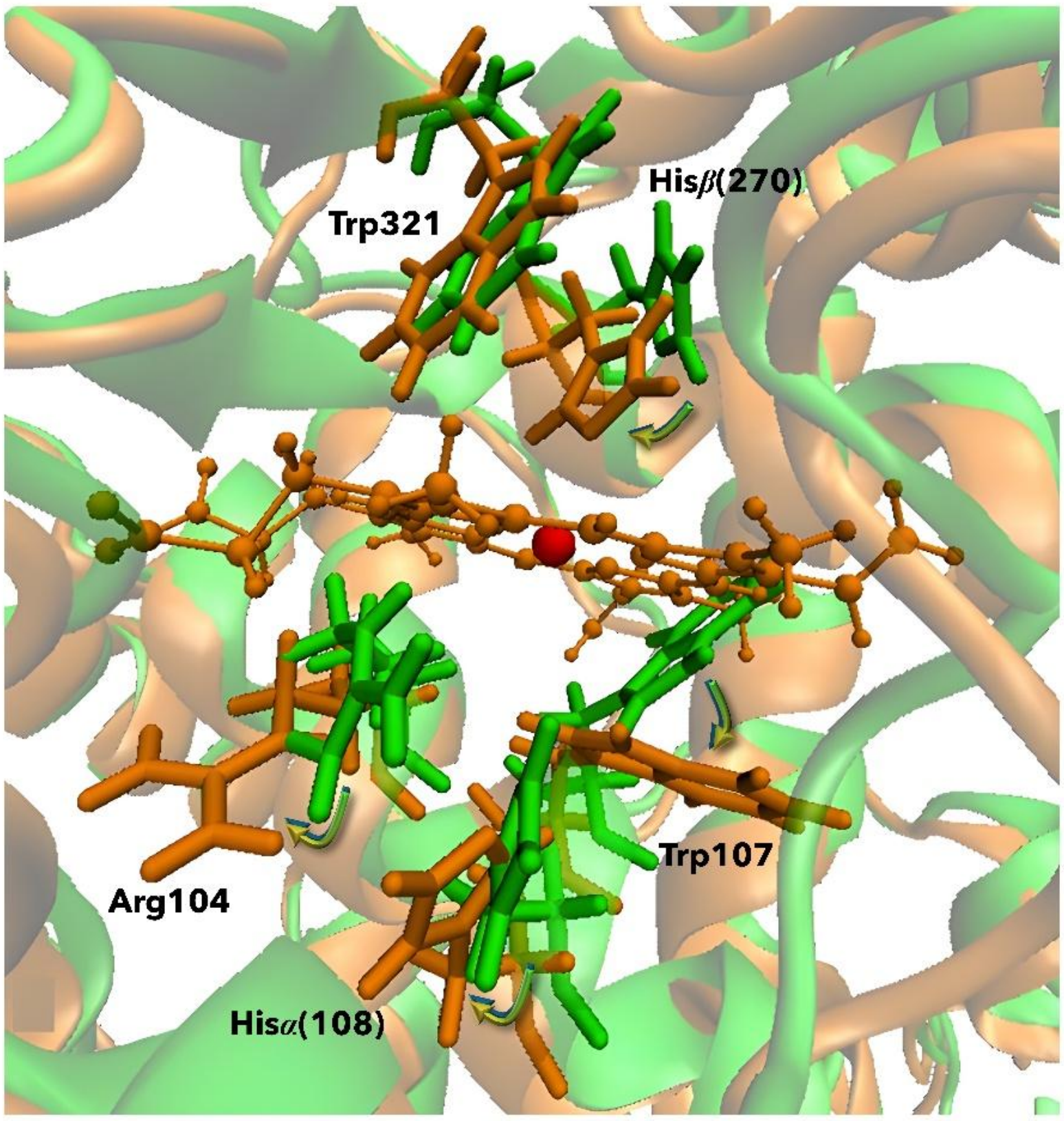

Figure S3. Effect of metalated heme B uptake. Superposition of the representative clustering-based MD structure of free KatG (green) with the crystal structure of Fe^{3+}-heme B bound KatG (orange; Fe^{3+} in red; PDB ID: 1SJ2). The arrows indicate the main displacements affecting the space occupied by heme B.

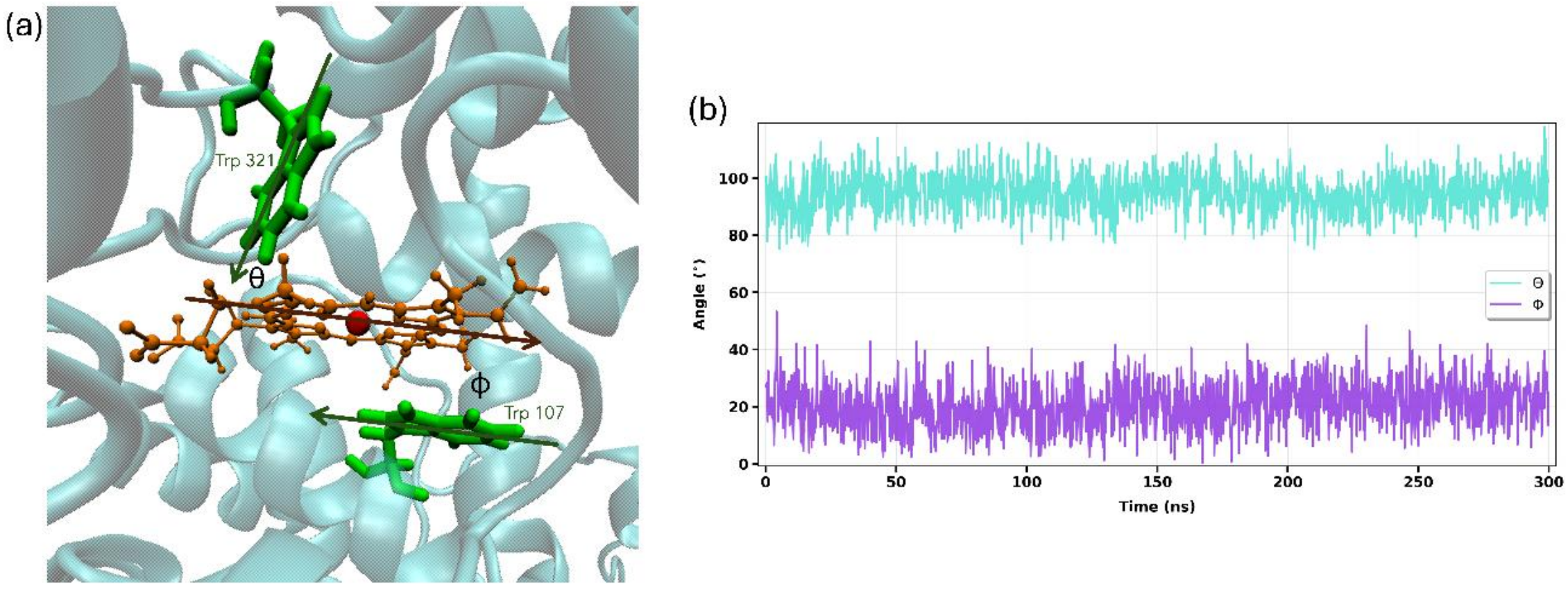

Figure S4. Orientation of Trp residues in the active site. (a) Vectors define the planar rings of the Trp107, Trp321, and the porphyrin of Fe^{3+}-heme B, forming angles ϕ (porphyrin–Trp107) and θ (porphyrin–Trp321). (b) Fluctuations of ϕ and θ along a representative MD trajectory are shown.

1. Computational Details

1.1 Quantum mechanical (QM) description of Fe^{3+}- and Ga^{3+}-heme B complexes

The unrestricted formalism of DFT was used for Fe^{3+}-heme B due to its sextet spin multiplicity, while Ga^{3+}-heme B corresponds to a singlet. Frequency calculations were performed upon the optimized geometries to verify that they correspond to minima on the potential energy surface and to obtain data for subsequent force-field parameterization. Concerning the calculation of partial atomic charges, the atomic radii of the metal centers must be explicitly defined, as they are not included in the standard procedure for calculating ESP charges. Therefore, Universal Force Field (UFF) radii were employed (Gaussian keyword: MKUFF), since they are available for the entire periodic table. In addition, the number of concentric layers of points and the density of points per layer were increased to 10 and 17, respectively (IOp(6/41=10) and IOp(6/42=17)).

1.2 Molecular dynamics (MD) simulations

1.2.1 **Force field parametrization**

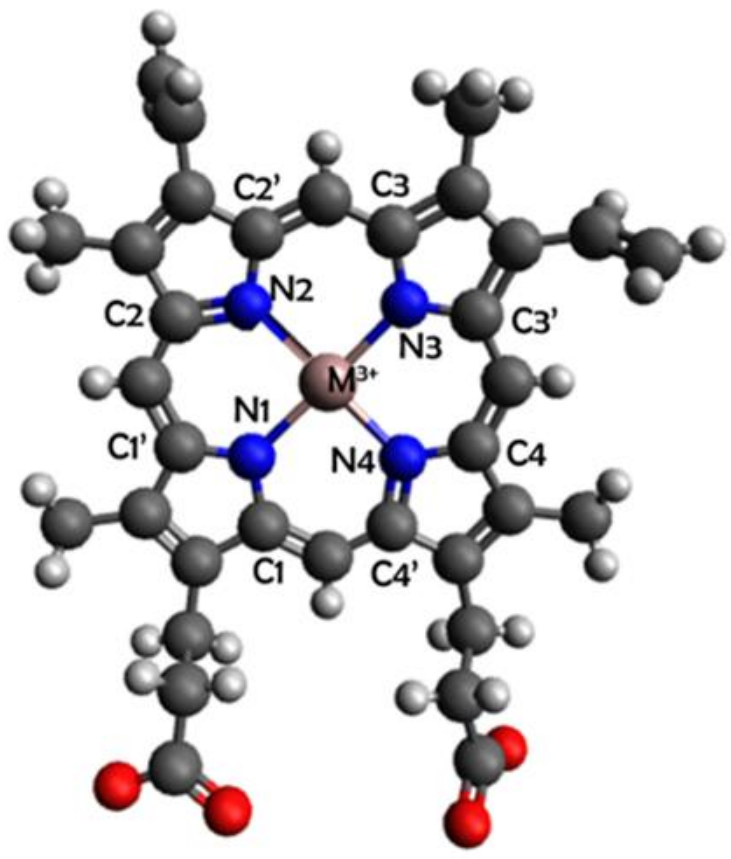

Figure S5. Atom types assigned to Fe^{3+}/Ga^{3+}-heme B complexes, derived from the MCPB.py procedure for force field parametrization.

Table S1. Force field parameters for the metal center. Parameters involve Fe^{3+} and Ga^{3+} within the heme B complexes, as defined in the MCPB.py workflow. A comparison with available crystallographic structures is given for bonds and angles.

**Charges** (*e*)			
**Fe^{3+}**	1.814009	**Ga^{3+}**	1.042886

**Bonds**							
**Type**	**Equilibrium value** (Å)	**X-ray value*** (Å)	**Force constant** (kcal/mol/ Å^2)	**Type**	**Equilibrium value** (Å)	**X-ray value**** (Å)	**Force constant** (kcal/mol/ Å^2)
**Fe-N1**	2.0374	2.04	83.0	**Ga-N1**	1.9898	2.00	88.2
**Fe-N2**	2.0521	2.08	80.2	**Ga-N2**	1.9955	2.02	88.9
**Fe-N3**	2.0486	2.07	79.0	**Ga-N3**	1.9944	2.01	84.9
**Fe-N4**	2.0430	2.04	81.6	**Ga-N4**	1.9896	2.03	92.3

**Angles**							
**Type**	**Equilibrium value** (°)	**X-ray value*** (°)	**Force constant** (kcal/mol/ rad^2)	**Type**	**Equilibrium value** (°)	**X-ray value**** (°)	**Force constant** (kcal/mol/ rad^2)
**N1-Fe-N2**	89.85	88.4	53.08	**N1-Ga-N2**	90.20	90.0	48.94
**N2-Fe-N3**	90.18	89.9	44.76	**N2-Ga-N3**	90.08	90.0	48.51
**N3-Fe-N4**	89.74	91.8	47.93	**N3-Ga-N4**	90.02	90.0	50.74
**N4-Fe-N1**	90.23	89.8	57.31	**N4-Ga-N1**	89.89	89.3	51.16
**N1-Fe-N3**	179.51	177.1	100.78	**N1-Ga-N3**	178.99	177.5	34.39
**N2-Fe-N4**	179.61	178.2	42.26	**N2-Ga-N4**	179.27	176.4	49.21
**Fe-N1-C1**	126.52	125.6	106.73	**Ga-N1-C1**	127.48	128.3	76.02
**Fe-N1-C1’**	126.64	127.5	104.38	**Ga-N1-C1’**	127.29	126.3	76.60
**Fe-N2-C2**	126.65	127.5	87.75	**Ga-N2-C2**	127.38	127.4	78.08
**Fe-N2-C2’**	126.12	125.9	98.73	**Ga-N2-C2’**	127.22	126.4	78.23
**Fe-N3-C3**	126.30	126.4	95.11	**Ga-N3-C3**	127.22	127.6	71.91
**Fe-N3-C3’**	126.65	125.3	91.12	**Ga-N3-C3’**	127.35	125.8	72.21
**Fe-N4-C4**	126.72	124.8	79.84	**Ga-N4-C4**	127.24	126.4	77.6
**Fe-N4-C4’**	126.19	127.4	89.02	**Ga-N4-C4’**	127.51	128.5	77.42

**Non-Bonded**					
**Type**	R_{vdw} (Å)	ϵ_i (kcal/mol)	**Type**	R_{vdw} (Å)	ϵ_i (kcal/mol)
**Fe^{3+}**	1.4000	0.01570749	**Ga^{3+}**	2.1915	0.41500000

*Crystallographic data obtained from: T. Bertrand, N. A. J. Eady, et al., ‘Crystal Structure of *Mycobacterium tuberculosis* Catalase-Peroxidase’, *J. Biol. Chem.*, **2004**, 279, p38991–38999. (PDB code: 1SJ2).

Crystallographic data obtained from: E. L. Dodd and D. Scott Bohle, ‘Orienting the heterocyclic periphery: a structural model for chloroquine’s antimalarial activity’, *Chem. Comm.*, **2014, 50, 13765–13768 (CCDC code: 904065).

1.2.2 **MD setup and production runs**

In all cases, the system was solvated using the tleap module of AmberTools22 with TIP3P[2] water molecules within a truncated octahedral box, ensuring a solvent shell of at least 8 Å from any solute

molecule. Due to the negative charge of these systems, K^+ ions were added to neutralize them. The protein was described using the ff19SB force field,[3] whereas the heme complexes through the *ab initio* parametrized force field described in the main text. The masses of heavy atoms were repartitioned[4] into the bonded hydrogens, thus slowing the highest-frequency motions and allowing for a longer time-step.

Each system was minimized by applying 2000 steps of the steepest-descent algorithm followed by 40,000 steps of the conjugate-gradient algorithm. It was then progressively heated for 1 ns from 100 K to 298 K in the NVT ensemble using a 1 fs timestep controlling the temperature by means of the Langevin thermostat[5] with a 1 ps^{-1} collision frequency. The bonds involving hydrogen atoms were constrained using the SHAKE algorithm.[6] Afterwards, the density was equilibrated with an isotropic position scaling through a 1 ns (1 fs timestep) simulation in the NPT ensemble, using Periodic Boundary Conditions (PBC). The temperature was controlled as in the heating process, and the pressure was kept at 1 bar by the Monte Carlo barostat.[7] The Particle Mesh Ewald (PME)[8] method was used with a grid spacing of 1 Å and non-bonded interactions in direct space were computed within a cutoff of 8 Å. The SHAKE algorithm was again applied. Finally, 300 ns of production were run using the same setup as in the density equilibration procedure, apart from the timestep and the collision frequency, increased to 4 fs and 5 ps^{-1}, respectively.

1.2.3 **Alchemical free energy calculations**

These simulations employ non-physical ("alchemical") intermediates to estimate the free energy associated with the gradual transformation of Fe^{3+} into Ga^{3+} during the simulation. A coupling parameter (λ) ranging from 0 to 1, was introduced such that when $\lambda = 0$ the potential energy surface corresponds to the pure Fe^{3+} system (initial state) and when $\lambda = 1$ it corresponds to the pure Ga^{3+} system (final state). For intermediate λ values, the system is a linear combination of the two states. Following previous studies[9–14] on heme-containing proteins, each alchemical transformation was simulated using 11 λ windows (0.0, 0.1, 0.2, 0.3, 0.4, 0.5, 0.6, 0.7, 0.8, 0.9, 1.0).

To avoid biasing the system toward either Fe^{3+} or Ga^{3+} configurations, the dimeric Fe^{3+} model was equilibrated corresponding to the half-path intermediate state (λ = 0.5). The atoms of the heme B

ligand coordinated to Fe^{3+}, together with the Fe^{3+} ion itself, were defined as the unique atoms of the initial state (λ = 0.0), whereas the atoms of the heme B ligand coordinated to Ga^{3+}, together with the Ga^{3+} ion itself, were defined as the unique atoms of the final state (λ = 1.0). To prevent the "end-point catastrophe," soft-core[15] potentials were applied to the disappearing Fe^{3+} and to the appearing Ga^{3+} ions for both van der Waals and electrostatic interactions, using Amber22 default parameters for the modified potentials in a one-step transformation. The system was then minimized for up to 10,000 steps using the steepest descent algorithm, followed by progressive heating and density equilibration, employing the same setup as in the MD simulations described above. The density-equilibrated system was then used as the starting point for the free energy calculations. For each of the 11 λ values, the system was minimized, heated, and density-equilibrated using the same protocol described above. Subsequently, a 15 ns production run was performed with a 2 fs time step and the resulting trajectories were post-processed to compute the free energy values according to the schemes described in the main text.

Concerning the three methods employed to compute the free energy changes (see main text), while the Thermodynamic Integration (TI) method estimates the free energy by analyzing the configuration data from each *λ* state independently, the BAR and MBAR methods evaluate data from pairs of adjacent λ states or from all λ states simultaneously, respectively.

1.2.4 **Hybrid quantum mechanics/molecular mechanics (QM/MM) calculations**

The structures used for the QM/MM simulations were selected based on a clustering analysis of the MD trajectories. Because bond formation between the metal center and nearby water molecules or amino acid residues is expected (as the MD simulations represent the encounter complex), only the trajectories of the Fe^{3+}-heme B systems (both within the protein and in explicit water) were analyzed, since in these simulations the metal remains closer to the axial residues. This approach avoids introducing any bias in the initial configurations between Fe^{3+} and Ga^{3+} models. Clustering was performed using the *k*-means algorithm, generating two clusters for the protein system, corresponding to the H-H and W-H configurations (see Fig. 3a). For the heme B system in explicit water, the optimal number of clusters was chosen as the one providing the best Davies–Bouldin Index (DBI), pseudo-F statistic (psF), and ssr/sst ratio.[16] The maximum number of iterations was set

to 500, with randomized initial centroids. The Root-Mean-Square Deviation (RMSD) among the atoms of the Fe^{3+}/Ga^{3+}-heme B complex, His108 and His270, was used as the distance metric for the protein system, whereas only the atoms of the Fe^{3+}/Ga^{3+}-heme B complex were considered when the complex was modeled in explicit water. One every four frames were sieved for clustering, and the remaining frames were subsequently assigned. The most representative frames for both the protein-bound and solvated complexes were then selected across all MD simulations by monitoring the DBI, psF, and ssr/sst parameters.

Cobramm2.0 contains the QM/MM scheme implemented in real space without employing Periodic Boundary Conditions (PBC). This approach poses a challenge when treating very large systems such as this protein complex. To address this, the system was reduced by cutting a 50 Å sphere centered on the QM region (the largest size allowed by the Cobramm2.0 software), while maintaining overall neutrality by adding an appropriate number of K^+ counterions and ensuring proper solvation. The atomic radii of the metal centers must be explicitly defined, as they are not included in the standard procedure for calculating the ChelpG charges required by COBRAMM2.0; therefore, UFF radii were used. The maximum step size for geometry optimization was set to 0.1 Bohr. During QM/MM geometry optimizations, the following default convergence criteria were applied: maximum force of 0.000450 a.u., root mean square (RMS) force of 0.000300 a.u., maximum displacement of 0.001800 a.u., RMS displacement of 0.001200 a.u.

1.2.6 **Force field validation**

To validate the force field, the Root Mean Square Deviations (RMSD) of the Fe^{3+}/Ga^{3+}-heme B complexes (using the QM optimized geometries as a reference) were plotted over a 300 ns MD simulation in explicit water (Fig. S6). The RMSD remains low throughout the simulation, and its fluctuations are mainly due to the free rotation of the carboxylate groups attached to the central porphyrin moiety.

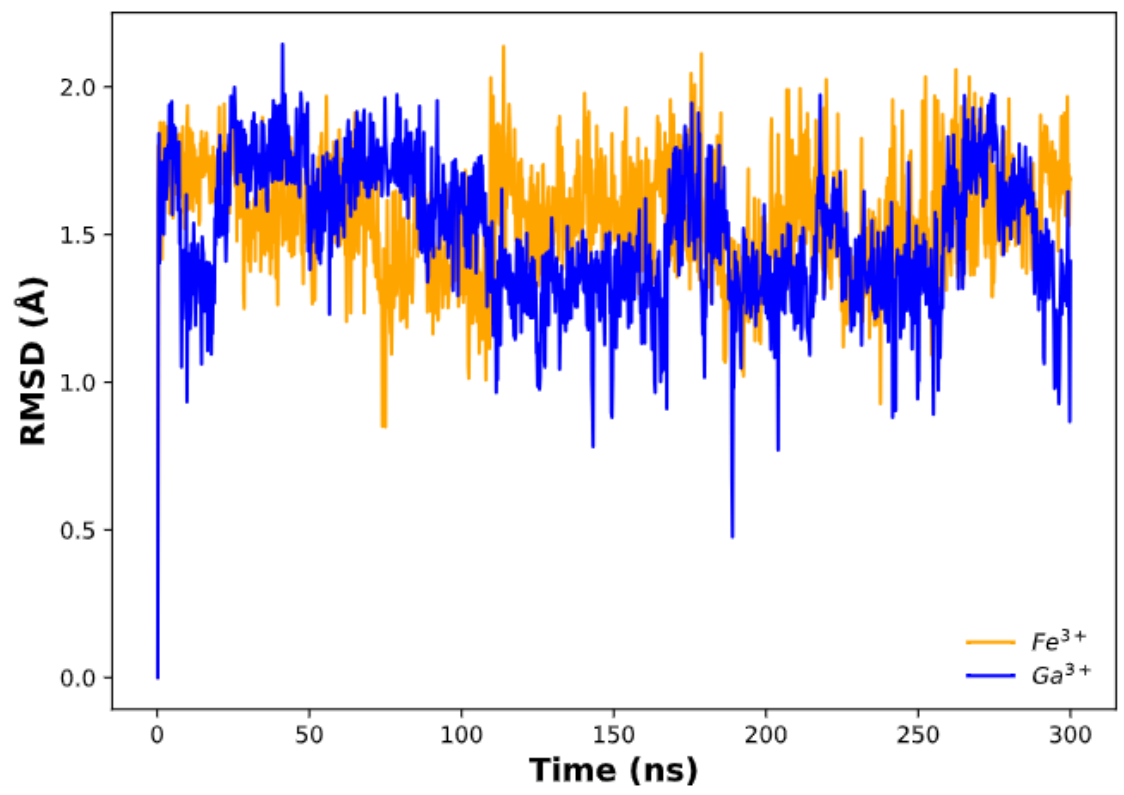

Figure S6. RMSD of the Fe^{3+}/Ga^{3+}-heme B complexes using the respective QM optimized geometry as a reference, during 300 ns MD simulation.

In addition, the ability of the parameterized force field to reproduce the QM-optimized geometry was tested. An MM-level geometry minimization was performed using an implicit solvent model[17] to ensure comparability with the QM geometry optimization. The superimposed structures are shown in Fig. S7 along with the RMSD value between the two. As can be seen, the structures obtained from QM and MM optimizations are almost perfectly superimposable, with a total RMSD between them consistently below 0.23 Å.

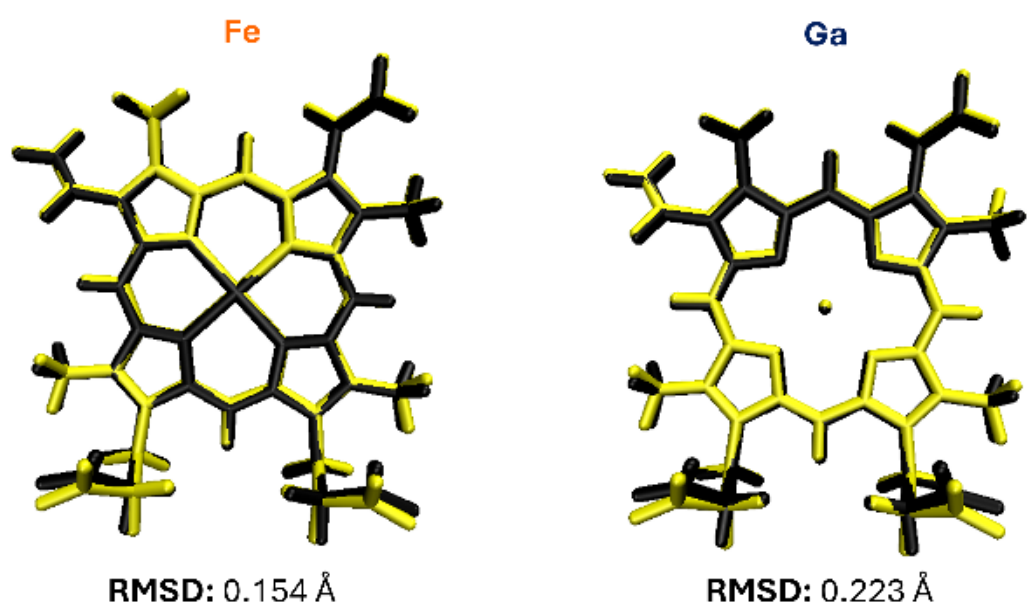

Figure S7. Superimposed (black: QM, yellow: MM) structures along with their RMSD difference.

To account for the difference in binding energy between protoporphyrin IX and the two metal ions, the free energies of protoporphyrin IX and of the isolated metal ions were calculated at the QM level. These values were then subtracted from the free energy of the corresponding metalloporphyrin complex. The resulting energy difference was divided by four to estimate the contribution of a single N–metal bond to the overall binding energy. The results are reported in Tab. S2:

Table S2. QM calculated binding energy between protoporphyrin IX and Fe^{3+}/Ga^{3+}, considering the contribution of a single N–metal bond to the overall binding energy

Bond	Binding Energy (kcal/mol)
N-Fe^{3+}	-34.8
N-Ga^{3+}	-107.7

The bond to Ga^{3+} is approximately three times stronger, consistent with the larger force constant and shorter equilibrium bond length reported for the Ga^{3+} force field (Table S1).

1.3 **Comparison with available QM data**

Table S3. Free energy data for the two possible substitution reactions associated with each binding mode shown in Fig. 3a of the main text. ΔG_{SMD} refers to the QM reaction modeled in implicit solvent (SMD: solvation model based on density), including the metallated heme B and the two ligands coordinated to the metal center (Histidine or water). ε denotes the static dielectric constant. $\Delta G_{QM/MM}$ refers to the QM/MM reaction modeled in explicit solvent (Figure 7 of the main text).

Reaction	ΔG_{SMD} (kcal/mol) (*)	$\Delta G_{QM/MM}$ (kcal/mol)
H-H@Fe → H-H@Ga	-14.9 (ε=4)<br>-21.7 (ε=30)<br>-25.7 (ε=78)	-3.5
W-H@Fe → W-H@Ga	-14.8 (ε=4)<br>-22.6 (ε=30)<br>-26.7 (ε=78)	-21.2

* Data obtained from reference 18 of the main text.

2. **Entropic contribution**

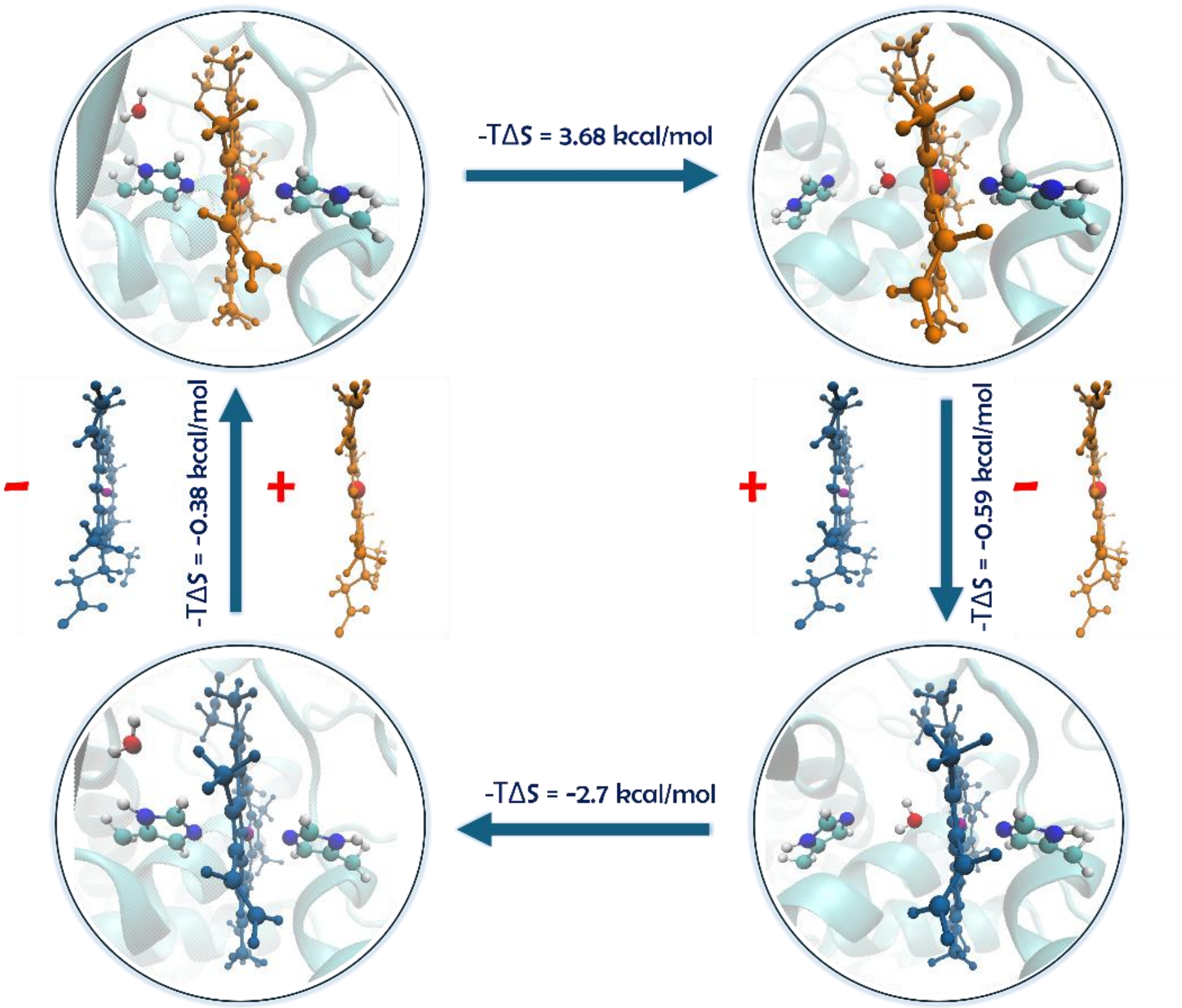

Figure S8. QM/MM entropic contributions (-TΔS) to the binding mode specific thermodynamics of gallium incorporation in KatG at room temperature, T = 298.15 K (see Figure 7 in the main text for the Gibbs free energy changes). H-H@Fe (top left); H-H@Ga (bottom left); W-H@Fe (top right); W-H@Ga (bottom right).

3. **Accuracy of the alchemical transformation**

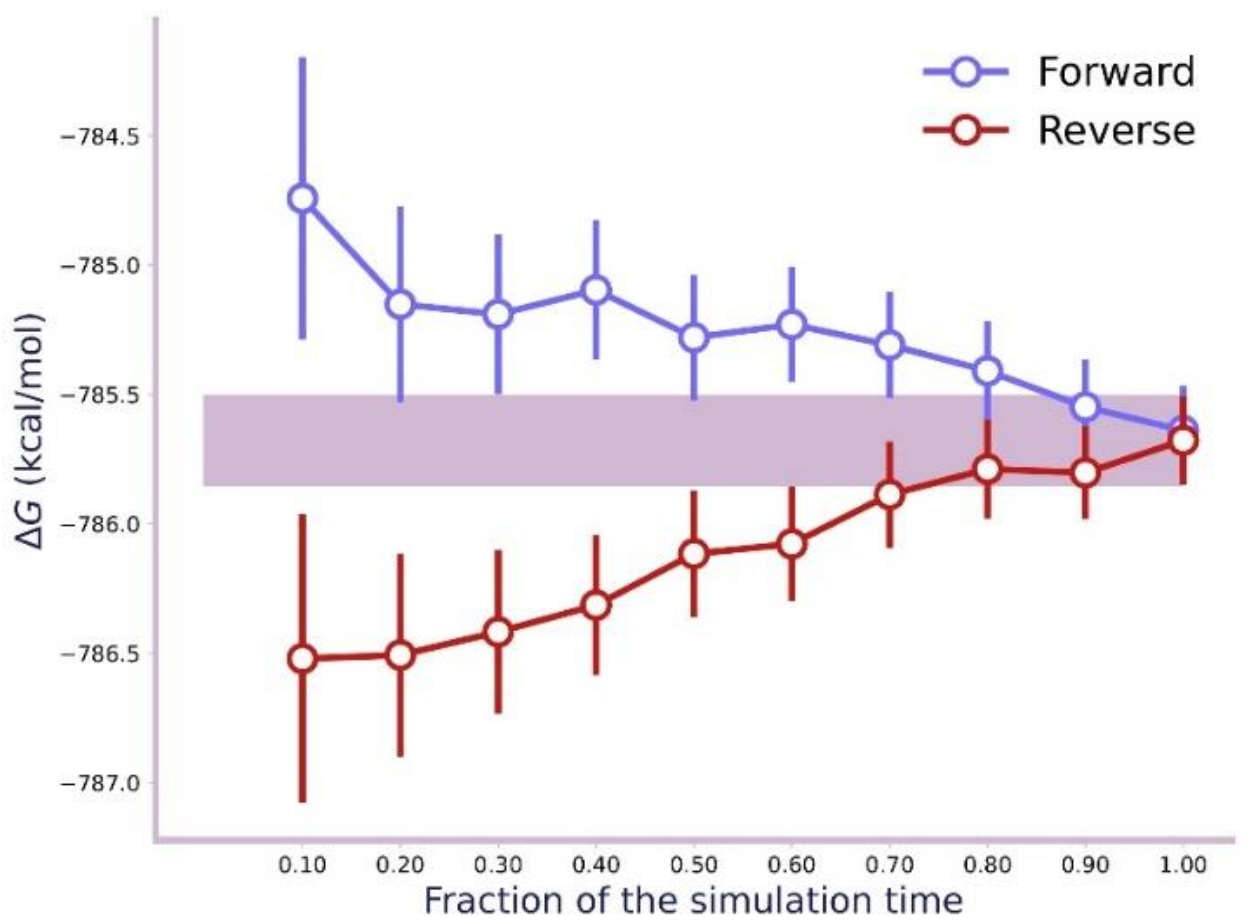

Figure S9. Convergence assessment of the alchemical transformation Fe^{3+}-heme B ⟶ Ga^{3+}-heme B embedded in the protein.

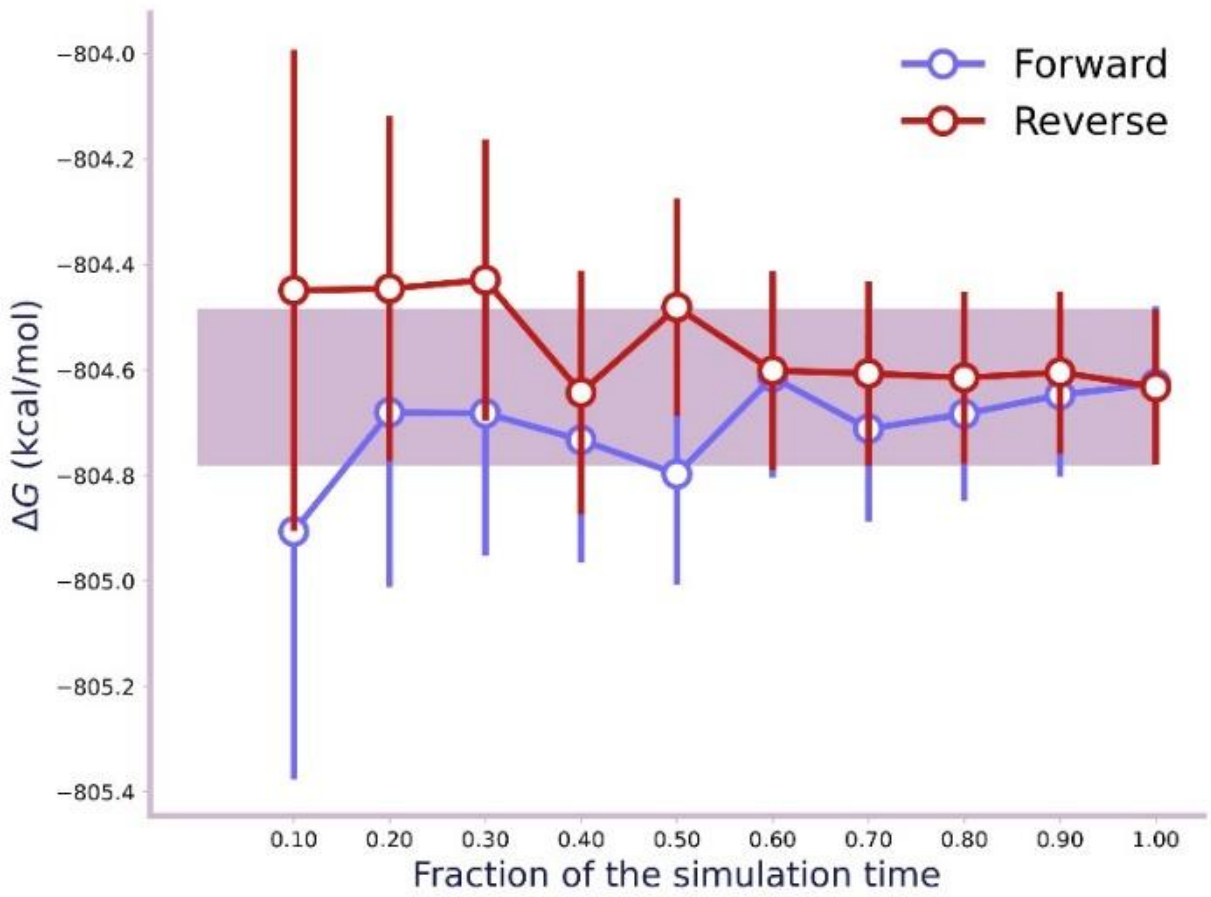

Figure S10. Convergence assessment of the alchemical transformation Fe^{3+}-heme B ⟶ Ga^{3+}-heme B embedded in explicit water.

The alchemlyb library (ref. 50 of the main text) provides estimates of the forward and backward free-energy convergence, which can be used to assess whether the simulation time was sufficient to achieve reliable sampling. For the transformation in explicit water, the convergence is excellent, as the error bars associated with the forward and reverse free-energy estimates overlap even when only a small fraction of the simulation data is considered. This behavior is expected given the simplicity of the environment surrounding the transforming species.

In contrast, convergence is more challenging for the transformation within the enzyme active site due to the increased complexity of the local environment and the possibility of interchanging binding patterns. Nevertheless, the error bars of the forward and reverse free-energy estimates overlap when approximately 80% of the simulation data is included. Furthermore, the difference between the two estimates at lower fractions of the simulation time remains below 2 kcal/mol. Overall, this analysis supports the reliability of the alchemical transformation protocol and indicates that the simulations were sufficiently converged for the purposes of this study.

For converged simulations, it is recommended a minimum overlap of 0.03 between neighboring states.[18] As reported in Figure S11, such overlap is guaranteed throughout the simulation.

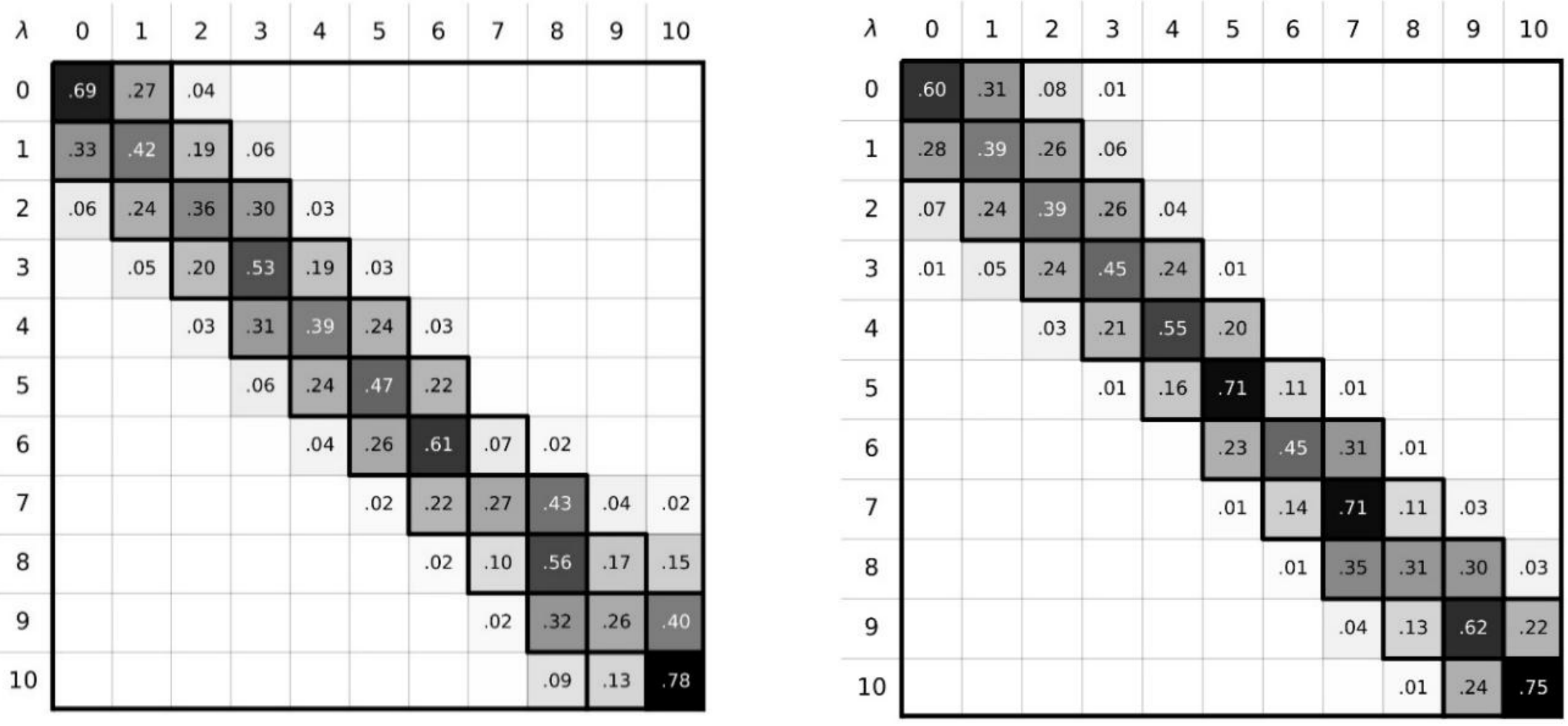

Figure S11. MBAR based overlap matrix for the alchemical transformation within the protein active site (left) and in explicit water (right), including all λ windows.

The effective sample size (Table S4) is the estimated number of statistically independent (uncorrelated) samples contained within a set of correlated molecular dynamics frames. Each λ window has 750 frames, and the effective sample size is a subset of these frames. Both using dU/dλ (TI) and u_{nk} (BAR, MBAR) observables for autocorrelation, the number of uncorrelated samples remains always higher than 50, the alchemlyb default threshold for a safe strict decorrelation.

Table S4. Effective sample sizes for each state based on the dU/dλ or u_{nk} observables for the transformation within the protein active site and in explicit water.

State index	Protein active site		Explicit water	
	**Effective sample size (dU/dλ)**	**Effective sample size (u_{nk})**	**Effective sample size (dU/dλ)**	**Effective sample size (u_{nk})**
0	651	662	444	423
1	552	536	478	460
2	391	427	550	499
3	659	646	551	535
4	277	390	660	617
5	372	380	750	750

6	322	319	349	343
7	98	94	743	750
8	395	388	224	233
9	222	209	415	564
10	310	644	611	531

The uncertainty estimates for each individual transformation, i.e., for each λ window (Table S5), indicate minimal errors in the 0.02 – 0.06 kcal/mol range.

Table S5. Uncertainty estimates for each individual transformation, including MBAR, BAR, and TI alchemical transformations.

**Transformation**	**ΔG_{MBAR} (kcal/mol)**	**ΔG_{BAR} (kcal/mol)**	**ΔG_{TI} (kcal/mol)**
0 → 1	0.06 ± 0.02	0.04 ± 0.03	0.05 ± 0.03
1 → 2	0.13 ± 0.03	0.15 ± 0.03	0.15 ± 0.03
2 → 3	0.14 ± 0.03	0.15 ± 0.03	0.16 ± 0.03
3 → 4	0.03 ± 0.03	0.06 ± 0.03	0.06 ± 0.03
4 → 5	-0.45 ± 0.04	-0.46 ± 0.04	-0.40 ± 0.04
5 → 6	-1.06 ± 0.04	-1.08 ± 0.05	-1.06 ± 0.04
6 → 7	-1.27 ± 0.06	-1.20 ± 0.06	-1.06 ± 0.05
7 → 8	-1.40 ± 0.04	-1.36 ± 0.04	-1.27 ± 0.05
8 → 9	-2.28 ± 0.03	-1.73 ± 0.05	-1.64 ± 0.05
9 → 10	-3.39 ± 0.03	-3.54 ± 0.03	-3.11 ± 0.04

4. **MD analysis**

The Root Mean Square Deviation (RMSD) of the active site is shown for each MD trajectory for monomer 1 (Fig. S12) and monomer 2 (Fig. S13). RMSD calculations included the metal ion, heme B, and the side chains of Arg104, Trp107, His108, His270, and Trp321 in monomer 1, corresponding to Arg844, Trp847, His848, His1010, and Trp1061 in monomer 2.

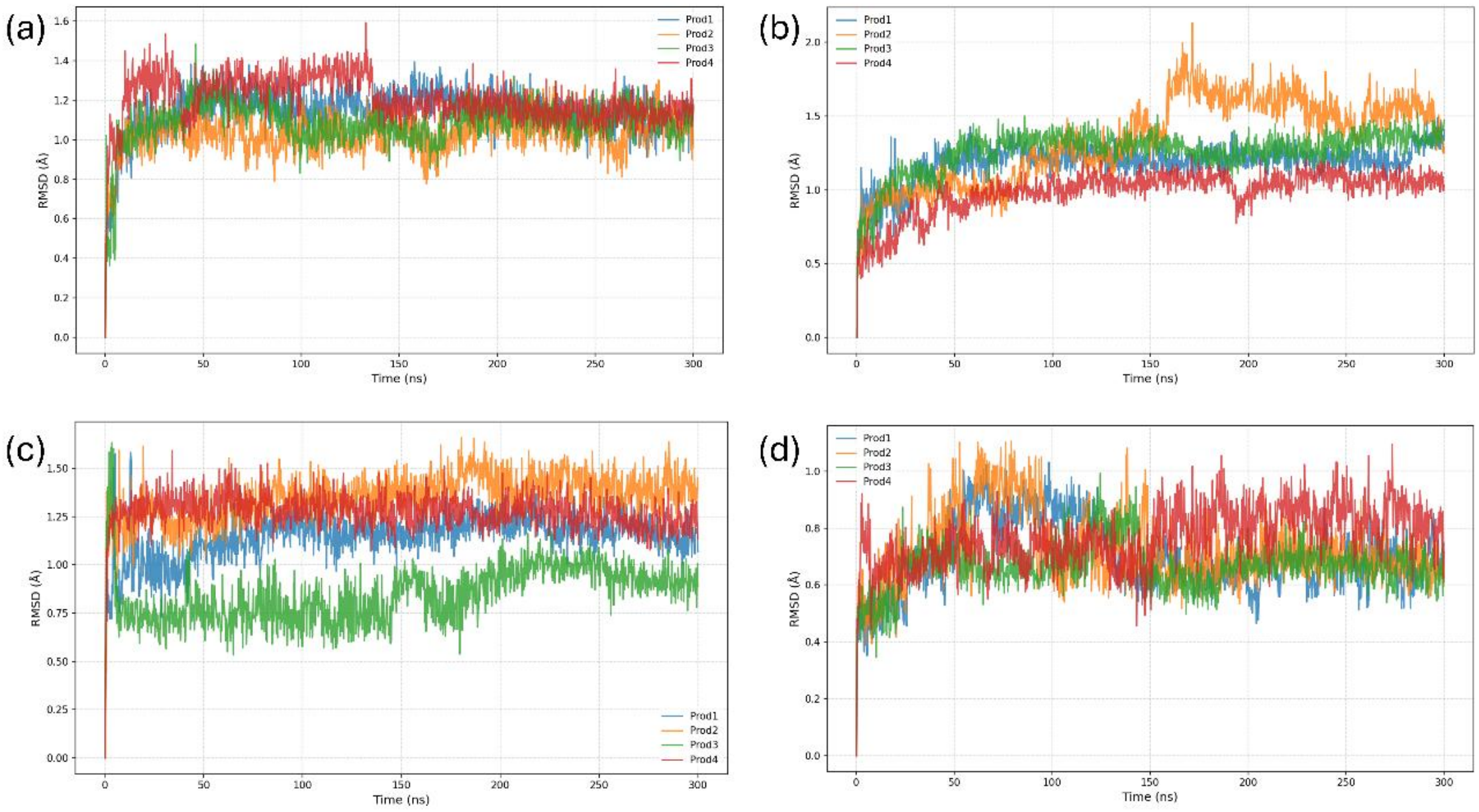

Figure S12. Root Mean Square Deviation (RMSD) of the active site in monomer 1. Each panel includes the analysis of four independent production runs where the metal ion is (a) Fe^{3+}, (b) Ga^{3+}, (c) Fe^{3+}, starting from the equilibrated Ga^{3+} model, (d) Ga^{3+}, starting from the equilibrated Fe^{3+} model.

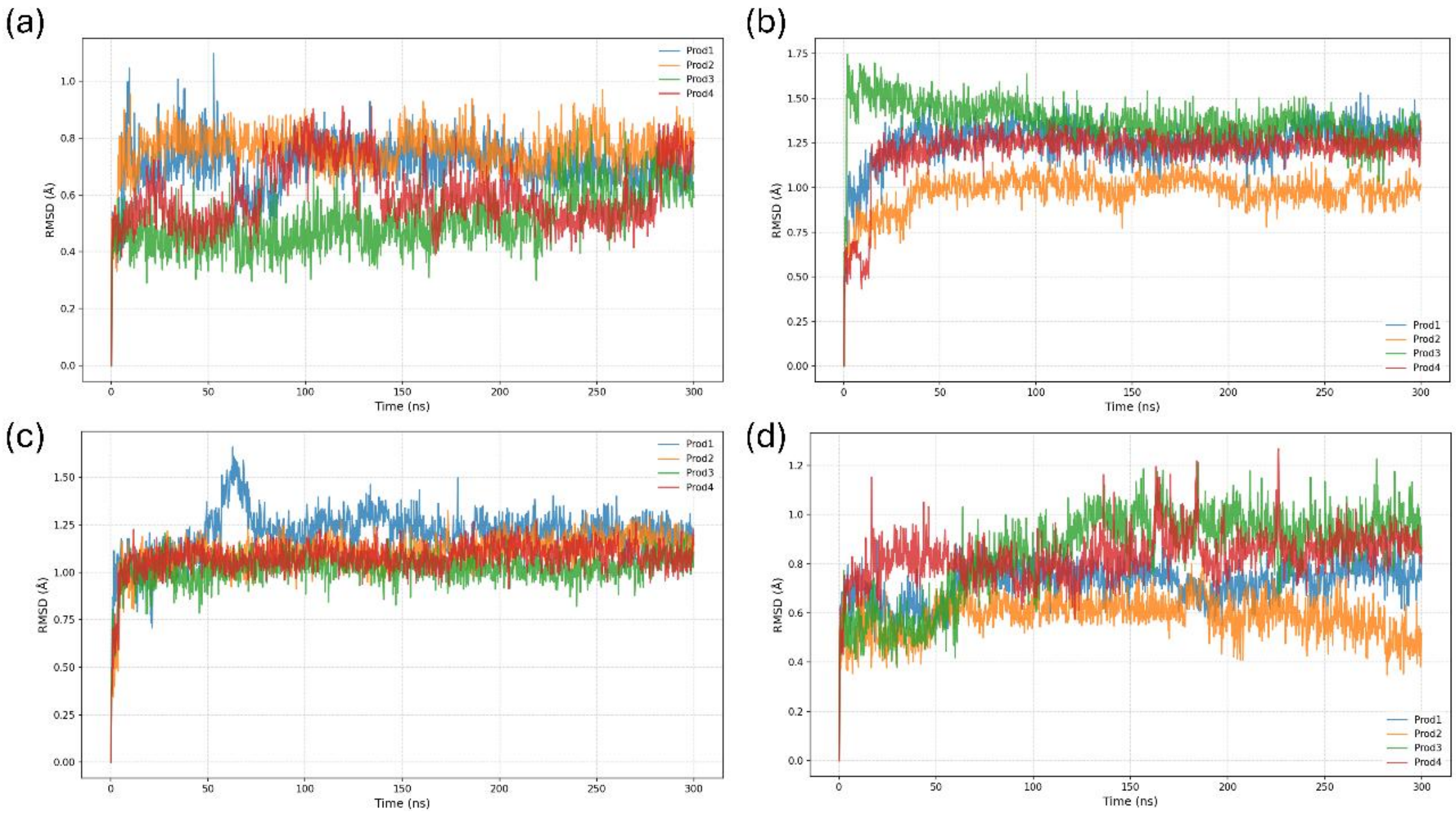

Figure S13. Root Mean Square Deviation (RMSD) of the active site in monomer 2. Each panel includes the analysis of four independent production runs where the metal ion is (a) Fe^{3+}, (b) Ga^{3+}, (c) Fe^{3+}, starting from the equilibrated Ga^{3+} model, (d) Ga^{3+}, starting from the equilibrated Fe^{3+} model.

5. **MSA analysis**

Multiple sequence alignments were visualized and analyzed using the NCBI Multiple Sequence Alignment (MSA) Viewer, Version 1.26.0 (National Center for Biotechnology Information, 2024), integrated within the NCBI BLAST environment (NCBI Resource Coordinators, 2018).

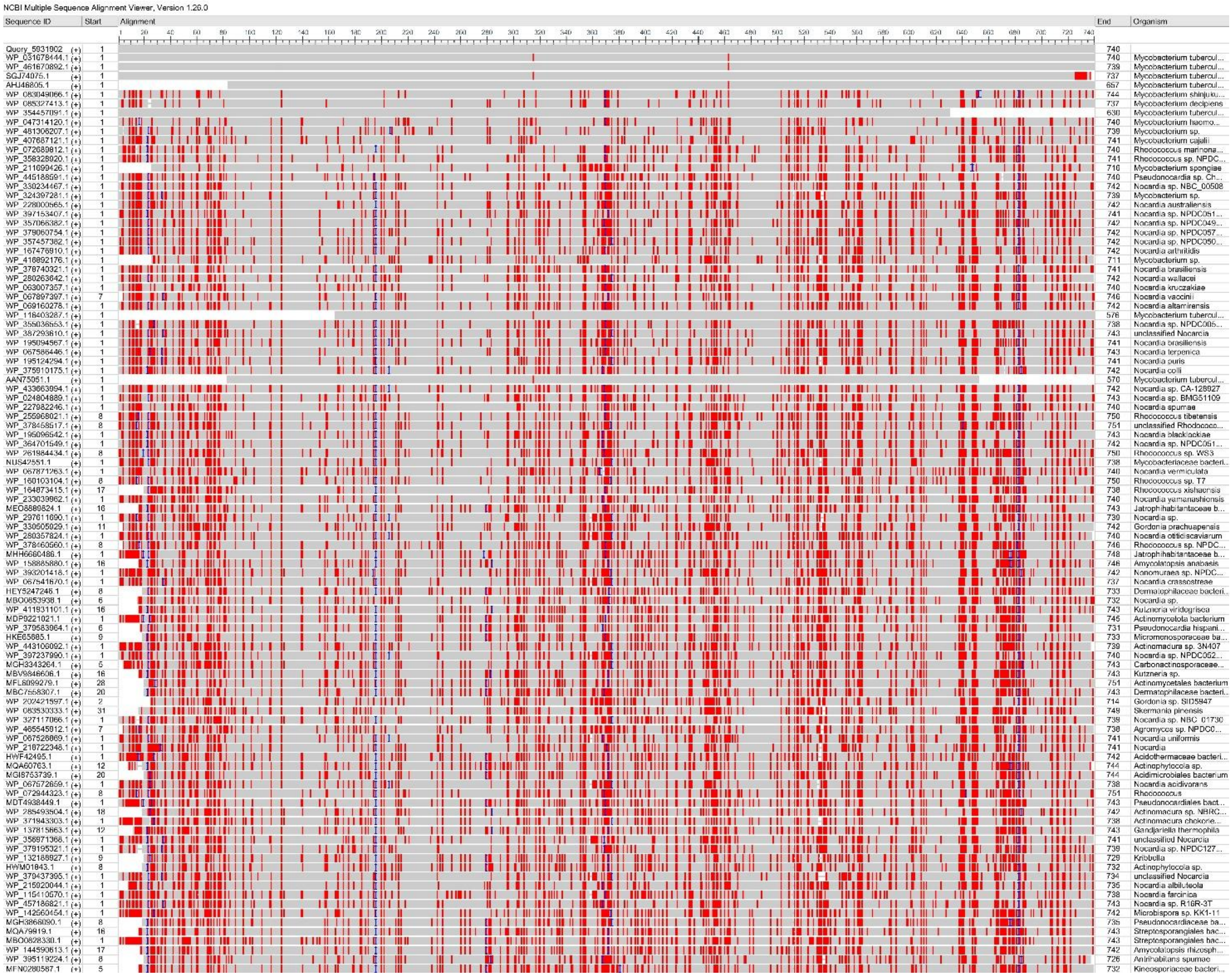

Figure S14. Multiple sequence alignments for the whole KatG monomer (residues 1-740).

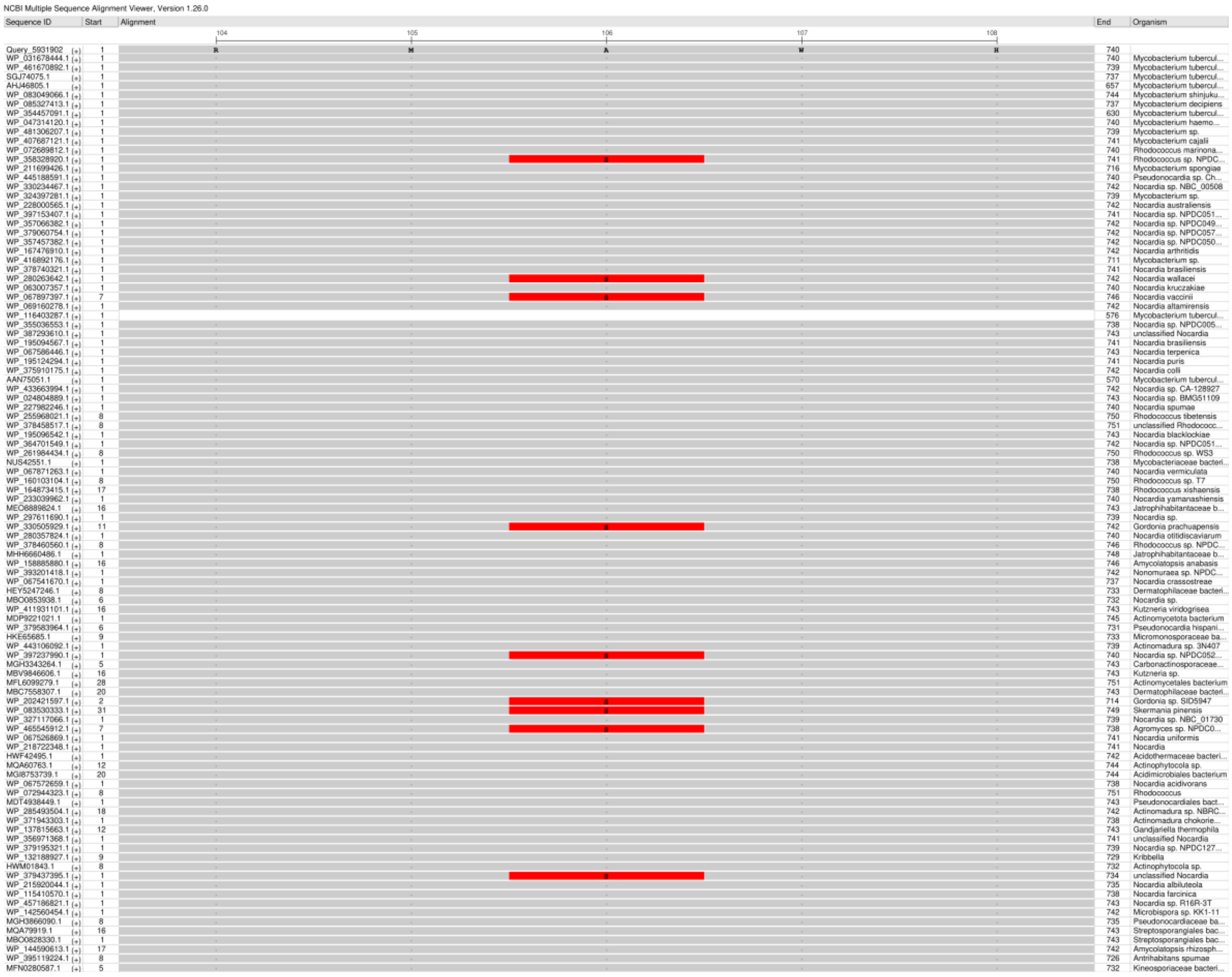

Figure S15. Multiple sequence alignments in the residues range: 104 – 108.

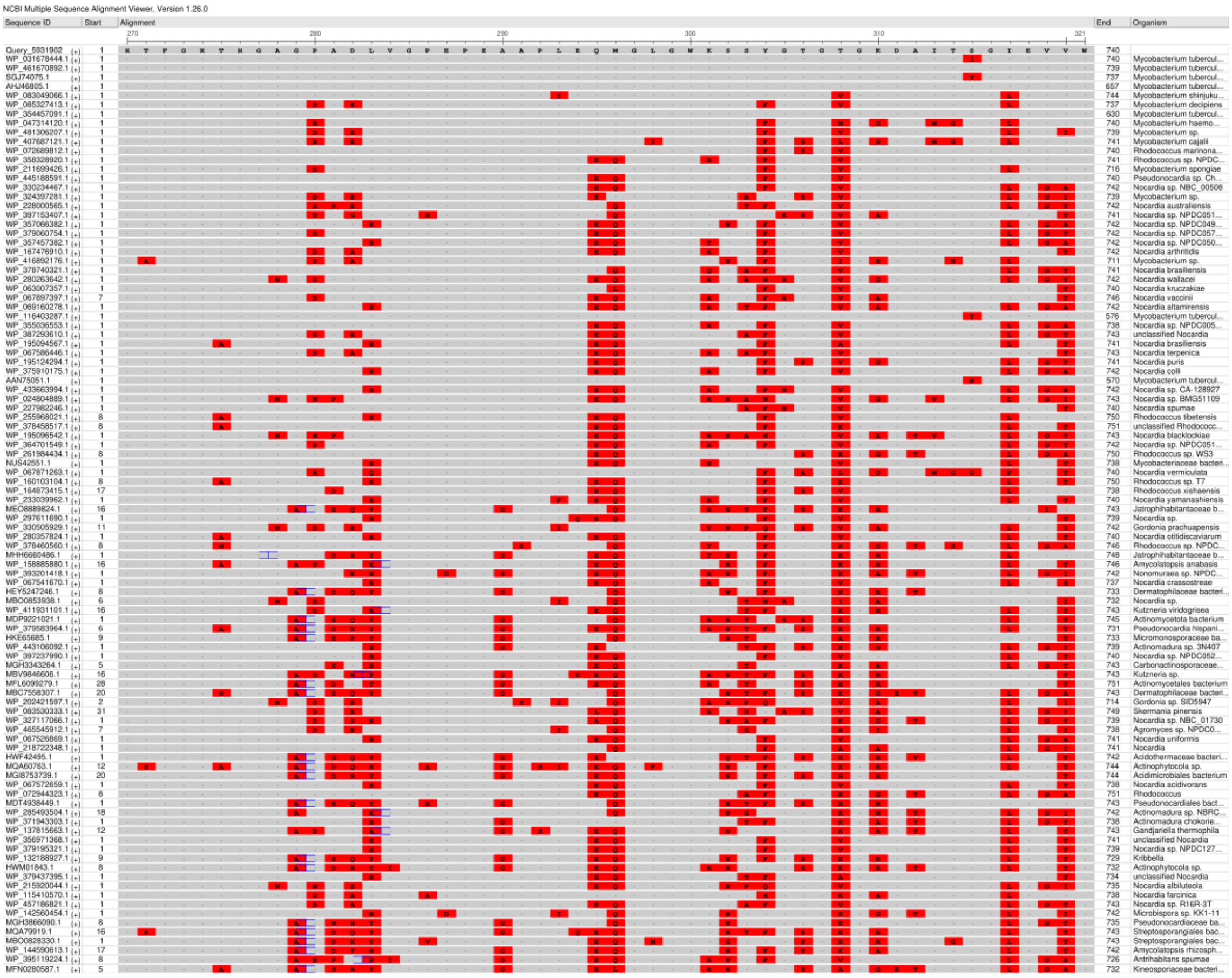

Figure S16. Multiple sequence alignments in the residues range: 270 – 321.

References

(1) *https://alphafold.ebi.ac.uk/entry/P9WIE5*.

(2) Jorgensen, W. L.; Chandrasekhar, J.; Madura, J. D.; Impey, R. W.; Klein, M. L. Comparison of Simple Potential Functions for Simulating Liquid Water. *J. Chem. Phys.* **1983**, *79*, 926–935. https://doi.org/10.1063/1.445869

(3) Tian, C.; Kasavajhala, K.; Belfon, K. A. A.; Raguette, L.; Huang, H.; Migues, A. N.; Bickel, J.; Wang, Y.; Pincay, J.; Wu, Q.; Simmerling, C. Ff19SB: Amino-Acid-Specific Protein Backbone Parameters Trained against Quantum Mechanics Energy Surfaces in Solution. *J. Chem. Theory Comput.* **2020**, *16* (1), 528–552. https://doi.org/10.1021/acs.jctc.9b00591

(4) Hopkins, C. W.; Le Grand, S.; Walker, R. C.; Roitberg, A. E. Long-Time-Step Molecular Dynamics through Hydrogen Mass Repartitioning. *J. Chem. Theory Comput.* **2015**, *11* (4), 1864–1874. https://doi.org/10.1021/ct5010406

(5) Schneider, T.; Stoll, E. Molecular-Dynamics Study of a Three-Dimensional One-Component Model for Distortive Phase Transitions. *Phys. Rev. B* **1978**, *17* (3), 1302. https://doi.org/10.1103/PhysRevB.17.1302

(6) Miyamoto, S.; Kollman, P. A. Settle: An Analytical Version of the SHAKE and RATTLE Algorithm for Rigid Water Models. *J. Comput. Chem.* **1992**, *13* (8), 952–962. https://doi.org/10.1002/jcc.540130805

(7) Metropolis, N.; Ulam, S. The Monte Carlo Method. *J. Am. Stat. Assoc.* **1949**, *44* (247), 335–341. https://doi.org/10.1080/01621459.1949.10483310

(8) Darden, T.; York, D.; Pedersen, L. Particle Mesh Ewald: An N Log (N) Method for Ewald Sums in Large Systems. *Journal of chemical physics* **1993**, *98*, 10089. https://doi.org/10.1063/1.464397

(9) Burggraf, F.; Koslowski, T. Charge Transfer through a Cytochrome Multiheme Chain: Theory and Simulation. *Biochimica et Biophysica Acta (BBA) - Bioenergetics* **2014**, *1837* (1), 186–192. https://doi.org/10.1016/j.bbabio.2013.09.005

(10) Diamantis, P.; Hage, K. El; Meuwly, M. Effect of Single-Point Mutations on Nitric Oxide Rebinding and the Thermodynamic Stability of Myoglobin. *J. Phys. Chem. B* **2019**, *123* (9), 1961–1972. https://doi.org/10.1021/acs.jpcb.8b11454

(11) Bauß, A.; Langenmaier, M.; Strittmatter, E.; Plattner, D. A.; Koslowski, T. Thermodynamic Integration Networks and Their Application to Charge Transfer Reactions within the AauDyPI Fungal Peroxidase. *J. Phys. Chem. B* **2016**, *120* (22), 4937–4944. https://doi.org/10.1021/acs.jpcb.6b03327

(12) Dumas, V. G.; Defelipe, L. A.; Petruk, A. A.; Turjanski, A. G.; Marti, M. A. QM/MM Study of the C—C Coupling Reaction Mechanism of CYP121, an Essential Cytochrome P450 of Mycobacterium Tuberculosis. *Proteins: Structure, Function, and Bioinformatics* **2014**, *82* (6), 1004–1021. https://doi.org/10.1002/prot.24474

(13) Poulos, T. L.; Kim, J. S.; Murarka, V. C. Computational Analysis of the Tryptophan Cation Radical Energetics in Peroxidase Compound I. *JBIC Journal of Biological Inorganic Chemistry* **2022**, *27* (2), 229–237. https://doi.org/10.1007/s00775-022-01925-8

(14) Xia, S.; Hirao, H. Stereo- and Regioselective Metabolism of the Omeprazole Enantiomers by CYP2C19: Insights from Binding Free Energy and QM/MM Calculations in a Curtin–Hammett Framework. *ACS Catal.* **2025**, *15* (4), 3284–3294. https://doi.org/10.1021/acscatal.4c07394

(15) Steinbrecher, T.; Mobley, D. L.; Case, D. A. Nonlinear Scaling Schemes for Lennard-Jones Interactions in Free Energy Calculations. *J. Chem. Phys.* **2007**, *127* (21), 214108. https://doi.org/10.1063/1.2799191

(16) Shao, J.; Tanner, S. W.; Thompson, N.; Cheatham, T. E. Clustering Molecular Dynamics Trajectories: 1. Characterizing the Performance of Different Clustering Algorithms. *J. Chem. Theory Comput.* **2007**, *3* (6), 2312–2334. https://doi.org/10.1021/ct700119m

(17) Onufriev, A.; Bashford, D.; Case, D. A. Exploring Protein Native States and Large-Scale Conformational Changes with a Modified Generalized Born Model. *Proteins: Structure, Function, and Bioinformatics* **2004**, *55* (2), 383–394. https://doi.org/https://doi.org/10.1002/prot.20033

(18) Klimovich, P. V; Shirts, M. R.; Mobley, D. L. Guidelines for the Analysis of Free Energy Calculations. *J. Comput. Aided. Mol. Des.* **2015**, *29* (5), 397–411. https://doi.org/10.1007/s10822-015-9840-9
